# Facile synthesis of silver nanoparticles using *Calotropis procera* leaves: unraveling biological and electrochemical potentials

**DOI:** 10.1186/s11671-024-04090-w

**Published:** 2024-09-03

**Authors:** Pooja V. Nagime, Nishat M. Shaikh, Sohel B. Shaikh, Chandrakant D. Lokhande, Vinod V. Patil, Sheeba Shafi, Dwi Marlina Syukri, Vijay R. Chidrawar, Ashwini Kumar, Sudarshan Singh

**Affiliations:** 1 Centre of Excellence in Innovative Biotechnology for Sustainable Utilization of Bioresources, Faculty of Agro-Industry, Prince of Songkla University, Hat Yai, 90110 Thailand; 2 Department of Biotechnology, Dayanand Science College, Latur, 413512 India; 3 Department of Medical Physics, Centre for Interdisciplinary Research, D. Y. Patil Education Society, Deemed to Be University, Kolhapur, 416006 India; 4 School of Chemical Sciences, Punyashlok Ahilyadevi Holkar, Solapur University, Solapur, 413255 India; 5 Department of Nursing, College of Applied Medical Sciences, King Faisal University, 31982 Al-Ahsa, Saudi Arabia; 6 Faculty of Medicine, Universitas Malahayati, Bandar Lampung, Lampung 35153 Indonesia; 7 School of Pharmacy and Technology Management, SVKM’s Narsee Monjee Institute of Management Studies (NMIMS), Deemed-to-University, Green Industrial Park, TSIIC, Jadcherla, Hyderabad 509301 India; 8 Research and Development Cell, Department of Mechanical Engineering, School of Engineering and Technology, Manav Rachna International Institute of Research and Studies, Faridabad, 121003 Haryana India; 9 Office of Research Administration, Chiang Mai University, Chiang Mai, 50200 Thailand; 10 Faculty of Pharmacy, Chiang Mai University, Chiang Mai, 50200 Thailand

**Keywords:** Antimicrobial, Antibiotic resistance, *Calotropis procera*, Electrochemical capacitive device, Silver nanoparticles, Supercapacitor

## Abstract

- CPL-AgNPs exhibited improved biomimetic attributes.
- Antibiotic resistance against pathogens were challenged through use of CPL-AgNPs.
- Supercapacitor application of facile synthesized AgNPs for the first time demonstrated improved physical application.

**Supplementary Information:**

The online version contains supplementary material available at 10.1186/s11671-024-04090-w.

## Abstract (articelhighlights)

The alarming rise of pathogen antibiotic resistance presents a major global health challenge and demands a novel way to control the microbial infections. Simultaneously, nanotechnology has found numerous uses in electrical as well as electronic systems, including timing, filtering, power factor adaptation, and capacitors for energy storage. This work investigates the synthesis and characterization of a silver nanoparticle (AgNPs) utilizing *Calotropis procera* (CPL) leaf extract. The optimization of synthesis process and the reduction of nanoparticles (NPs) were validated by UV–visible spectroscopy. AgNPs' was exhaustively characterized for morphology, crystallinity, zeta-potential, and structural properties. The produced NPs demonstrated a wide range of characteristics, such as antioxidant, antidiabetic, antibacterial, and antifungal effects. Furthermore, remarkable electrochemical performance was indicated by the CPL-AgNPs electrode, which has mesoporous, clustered sphere-shaped particles onto a flexible stainless-steel substrate. This highlights the electrode's potential in energy storage applications. Copper monosulfide served as the anode and CPL-AgNPs as the cathode electrode in tested hybrid supercapacitor devices, which proved remarkable specific capacitances, high specific energy, and exceptionally high specific power. In order to address the twin challenges of antimicrobial resistance alongside advanced energy storage, this study provides a novel and thorough analysis of the basic electrochemistry as well biological properties of AgNPs, clarifying their potential storage of charges mechanisms and biomedical applications.

**Graphical abstract:**

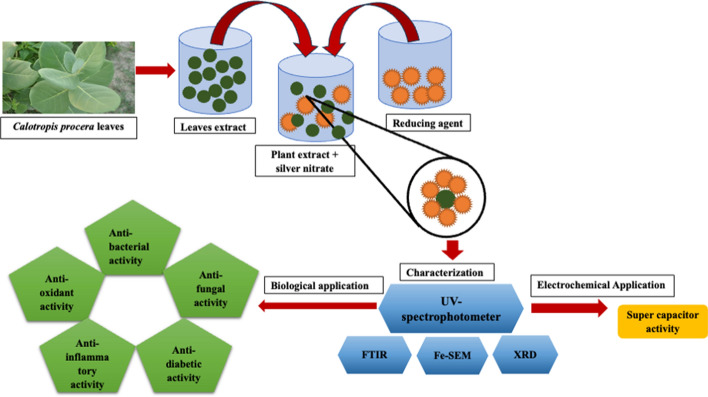

**Supplementary Information:**

The online version contains supplementary material available at 10.1186/s11671-024-04090-w.

## Introduction

Nanotechnology stands as one of the most promising fields for innovating electrical capacitors, biotechnology, medical, and surgical applications [1–3]. Over the years, nanoparticles (NPs) have undergone extensive research across various sectors including agriculture, biomedicine, surface treatment and coatings, food industry, and energy production [4]. Their unique nanoscale properties and nanostructures have sparked significant interest. Currently, the synthesis of nanoscale metals through biological, chemical, and physical approaches remains a focal point of extensive research. With challenges such as extreme energy consumption, high costs, time-intensive processes, hazardous conditions requiring high pressure and temperature, and emission of dangerous substances; however, green synthesis approaches are gradually replacing physical and chemical procedures due to the use of sophisticated equipment and synthesis conditions [5]. As an alternative, green synthesis approach does not involve the use of chemicals. Additionally, they are straightforward, single-step, eco-friendly, and stable. Microorganisms (bacteria, algae, fungi) and plant extracts serve as agents in green synthesis due to their reducing and antioxidant properties, facilitating the conversion of metallic compounds into NPs. Moreover, substances originating from sea as waste that are transformed to biomaterials are also reported as potential source that can reduce metallic NPs [6–8]. However, manufacturing NPs using microbes or spent microbial media is not feasible on an industrial scale due to the requirement for a highly aseptic environment and maintenance [9]. Researchers have particularly emphasized the use of green technology i.e. plant extracts to fabricate NPs due to their cost-effectiveness, nontoxic nature, ease of use, and ecological qualities. The phytochemicals present within plant extracts, including proteins, amino acids, sterols, flavonoids, alkaloids, phenolics, terpenoids, and others, act as stabilizing and reducing agents for the nanoparticles [10].

There are various types of metallic NPs such as gold [11, 12], silver [10, 13], copper, cadmium, iron, zinc, platinum, selenium [14], and more reported with biological applications [5]. The favorable characteristics of metal NPs include high surface area-to-volume ratios, strong reactivity, and consistent size distributions, making them highly attractive. Among these, AgNPs have garnered significant attention due to their numerous applications in the pharmaceutical and biomedical fields [13]. Over time, silver has emerged as a potent antibacterial agent, finding utility across a wide range of nanostructured materials in various sizes and shapes. Additionally, AgNPs exhibit excellent conductivity, chemical stability, localized surface plasmon resonance, and catalytic activity [15]. These NPs possess unique biological properties such as anti-inflammatory, anti-tumor, antibacterial, antifungal, antiparasitic, antiviral, and anti-tumor actions [16]. In dentistry and dental implants, AgNPs are employed for their therapeutic qualities in wound dressings, ventricular drainage catheters, catheters impregnated with silver, and in the prevention of orthopedic infections and osteointegration [17]. Silver exhibits potent antimicrobial properties by damaging the cell wall of bacteria, thereby inhibiting their growth, and disrupting their metabolism [18]. This occurs as Ag^+^ ions interact with proteins and DNA within bacterial cells, leading to inhibited protein synthesis, decreased membrane permeability, and ultimately, bacterial cell death. AgNPs, being considerably more chemically reactive than silver ions, serve as excellent antibacterial agents [19].

Beyond biomimetic attributes, a plethora of research has shown how important AgNPs are for improving the electrochemical characteristics of several types of electrode materials. Studies demonstrated that AgNPs composite exhibit specific capacitance about 138.2 F g^−1^ in presence of in 1 M H_2_SO_4_; however, an improvement in specific capacitance of 591 F g^−1^ was reported [20, 21]. In similar study AgNPs demonstrated a specific capacitance of 424 F g^−1^, a density of energy of 14.04 Wh kg^−1^, along with a power density of 6.41 kW kg^−1^ in super-capacitive experiments [22]. Whereas when AgNPs was used as material through 3D printer microfluidic device for hydrogen peroxide (H_2_O_2_) sensing and supercapacitor functions demonstrated an impressive 367.16 mF cm^−2^ storage capacity as a supercapacitor, with exceptional cyclic stability across 1500 charge–discharge cycles, at a current density of 1 mA cm^−2^ was observed. Moreover, three-electrode microfluidic system, the electrode for H_2_O_2_ sensing obtained a limit of detection of 0.52 µM throughout a linear range of 1–10 µM [23]. Therefore, these results indicate the potential of AgNPs in energy storage applications, which is also supported by some similar experimental analysis using inorganic metals such as zinc oxide [24], ceric oxide dopped nickel [25], iron dopped zinc oxide nanocrystals [26], and cadmium dopped zinc oxide electrodes [27].

*Calotropis procera* is a flowering plant belonging to the Asclepidaceae family, native to Saudi Arabia, North Africa, Pakistan, tropical Africa, Western Asia, South Asia, Israel, and Indo-China region [10]. The leaves of this plant contain several active chemicals such as toxic glycosides, calotropin, uscharin, and calotoxin. Moreover, the whole plant has been used traditionally as an anticoagulant and anticancer agent. In previous studies, it has been suggested that this plant exhibit anti-inflammatory, analgesic, and antioxidant properties [28]. It has been also used in the treatment of asthma, earaches, stomach aches, arthritis, and skin diseases. Additionally, the bioactive compounds present in the extract of C. *procera* have been further used to treat somatic, sinus, diarrhea, fistula, skin diseases, and jaundice [29]. Therefore, using *C. procera* leaf extract, this study employs a biological strategy to increase the efficiency of synthesizing AgNPs for biomimetic attributes and their use in an electromechanical supercapacitor. The primary objective of this research is to investigate a variety of possible highlights, such as antibacterial, antifungal, antioxidant, anti-inflammatory, and antidiabetic properties for AgNPs. Additionally, the study explores structural and morphological characterization, optimization methods, and possible electrochemical uses of the fabricated AgNPs.

## Materials and methods

### Materials

The experimental reagents used are of analytical grade such iodine (Mw. 126.9 g moL^−1^), sodium hydroxide (Mw. 39.997 g moL^−1^; 97% of purity), naphtha, copper acetate (Mw. 181.6 g moL^−1^), sulfuric acid, chloroform, glacial acetic acid, benzene, perchloric acid, ferric chloride (Mw. 162.2 g moL^−1^; 99% of purity), hydrochloric acid, 2,2-Diphenyl-1-picrylhydrazyl (DPPH; Mw. 394.32 g moL^−1^), hydrogen peroxide, ascorbic acid (Mw. 176.12 g moL^−1^), sodium nitroprusside (Mw. 261.92 g moL^−1^), nitric oxide (Mw. 30.01 g moL^−1^), butylated hydroxytoluene (BHT; Mw. 220.35 g moL^−1^), phosphate buffer, potassium ferricyanide (Mw. 329.24 g moL^−1^), trichloroacetic acid (TCA), α-amylase (Mw. 54000 dalton), dinitro salicylic acid (Mw. 228.12 g moL^−1^), nitrophenyl α-diglycopyraside (Mw. 301.25 g moL^−1^), α-glucosidase (Mw. 51 kDa), sodium carbonate (Mw. 105.98 g moL^−1^), dipeptidyl peptidase (Mw. 30 kDa), glypropnitroanilide (Mw. 328.75 g moL^−1^), bovine albumin (Mw. 66.5 kDa), aspirin (Mw. 180.15 g moL^−1^), lipoxidase, and linoleic (Mw. 280.44 g moL^−1^) acid were procured from Merck, Mumbai, India. Carbon black (C), polyvinylidene fluoride (PVDF; Mw. 530,000), N-methyl-2-pyrrolidone (NMP; Mw. 99.113 g moL^−1^), copper (II) sulfate (CuSO_4_; Mw. 159.6 g moL^−1^), triethylamine (TEA; Mw. 101.191 g moL^−1^), sodium thiosulfate (Na_2_S_2_O_3_; Mw. 158.11 g moL^−1^), and polyvinyl alcohol (PVA; Mw. 26300 g moL^−1^) procured from Sigma Aldrich, Sigma-Aldrich Chemicals Private Limited Bangalore, India. Additionally, stainless steel (304 grade) with a thickness of 0.25 mm was acquired from a local market in Kolhapur, India. Media such as nutrient agar and potato dextrose agar were used, along with bacterial *(Bacillus subtilis-*MTCC 121 T*, Bacillus megaterium-*NCTC 6094) and Gram-negative (*Shigella flexneri-*MTCC 1457) and fungal (*Trichoderma viride-*ITCC 1433*, Penicillium crysogenum*-NCPF 2802*,* and *Aspergillus niger*-MTCC 12975) microorganisms sourced from the Department of Microbiology, Dayanand Science College, Latur, India. All other chemicals used were of analytical grade.

### Methods

#### Plant collection and preparation of aqueous extract

Fresh *C. procera* leaves (CPL) were collected from a hygienic local area in Latur, Maharashtra, India, and authenticated by Dr. Hawaldar V Venkatrao at the Department of Botany, Dayanand Science College, Latur, India (Herbarium number B/DSC/013-2023). The leaves were removed from the stem and washed twice with double-distilled water and subsequently with 70% ethanol. Consequently, the leaves were dried at 60 °C in a vacuum oven and finely powdered using a grinder (Phillips Domestic Appliances India Ltd, Chennai, India). A 50 gm of the fine dried leaves powder was then transferred to a 1000 mL beaker containing 250 mL of distilled water, and the mixture was boiled at 100 °C for 20 min. After cooling to room temperature, the solution in the beaker was filtered using Whatman filter paper number 1. The filtrate was stored at 4 °C till further use.

#### Phytochemical screening

Phytochemical analysis was conducted to identify the presence of secondary metabolites such as alkaloids, anthocyanins, diterpenes, flavonoids, glycosides, phenols, phlobatannins, proteins, tannins, and terpenoids as reported [10].

#### Biosynthesis of AgNPs

**Fig. 1:**
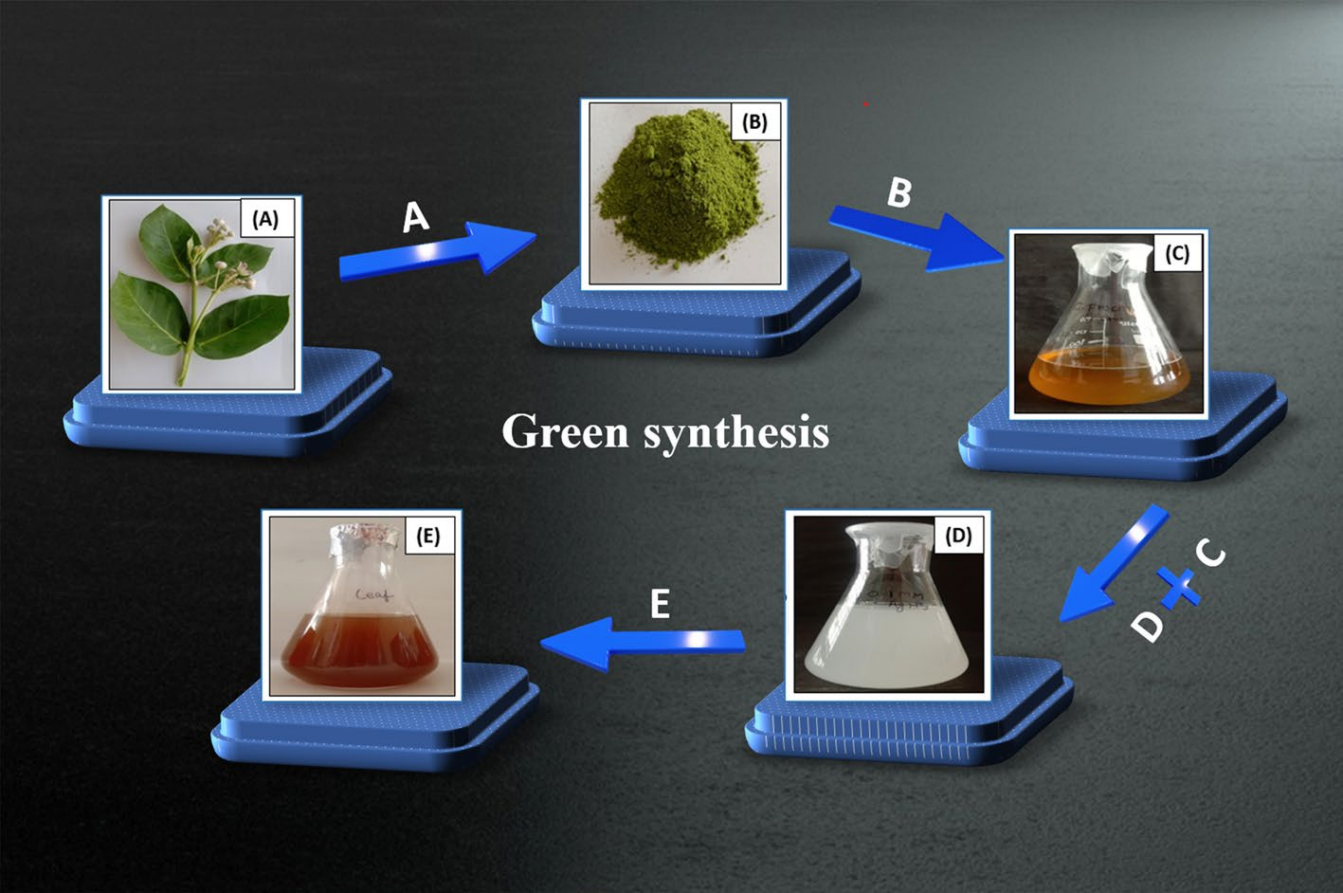
Green synthesis of AgNPs using CPL extract after incubation at 40 °C for 2 h: *C. procera* leaves (**A**); CPL powder (**B**); CPL extract after filtration (yellowish) (**C**); AgNO_3_ solution (colorless) (**D**); dark brown color as CPL-AgNPs (**E**)

Silver NPs were synthesized using a bottom-up approach as reported [10]. In brief, 4 mM aqueous AgNO_3_ solution (500 mL) was prepared, and the containing flask was refrigerated at 0 °C for 2 h. AgNPs were successfully synthesized at a 1:3 ratio of plant aqueous extract to AgNO_3_ solution (10–30 mL), with the plant extract serving as a reducing or capping agent and AgNO_3_ as a precursor. The optimized concentration of AgNO_3_ (4 mM) was used, and for the synthesis, 100 mL of extract was mixed with 300 mL of AgNO_3_ solution. The optimized conditions with respect to time and temperature were 40 °C for 4 h, respectively in an incubator was tested. A color change from colorless AgNO_3_ solution to dark brown confirmed the synthesis of AgNPs, validated through UV spectrophotometry. The pellets of AgNPs were obtained from the mixture by twice centrifugation (MC-12 Plus, Wasai, Maharashtra, India) at 15,000 rpm for 15 min. Post-centrifugation, the pellet was rinsed twice with distilled water to remove any remaining impurities and unreduced silver nitrate, followed by vacuum drying (HMG, Vasai East, Mumbai, India) at 60 °C and storage at room temperature for further characterization (Fig. 1). Various parameters that affect the synthesis of AgNPs, such as incubation time, reaction temperature, volume of extract, and concentration of silver ions, were further studied.

#### Characterization of silver nanoparticles

The UV–visible spectrometer (Agilent Technologies, Cary 60 UV–vis, Santa Clara, CA, USA) was used to study surface plasmon resonance (SPR) and optical properties were employed to characterize the AgNPs. Fourier-Transform Infrared Spectroscopy (FTIR) (Perkin Elmer L1600401, Middlewich, Cheshire, UK) was utilized to identify the functional groups to which silver ions are bonded. While X-ray diffraction (XRD) (XRD-7000 X-ray diffractometer, Shimadzu Corporation, Chiyoda-Ku, Tokyo, Japan) with Cu-Kα radiation at a wavelength of 1.5406 Å and scanning angle 2θ from 20–0 degrees was employed to identify the crystalline structure of synthesized AgNPs at the atomic level. Further, the d-spacing for the XRD analysis was calculated using Bragg's Law, which relates the wavelength of the incident X-rays, the diffraction angle, and the interplanar spacing (d-spacing) of the crystal using Equ. 1. Furthermore, the Debye–Scherrer equation was used to calculate the size of synthesized AgNPs using below given Equ. 2.

1 $$\text{n}\lambda = 2dsin\theta$$

where *n* is the order of reflection (which is 1 in this case), λ is the wavelength of the X-ray (1.5406 Å for Cu-Kα radiation), and θ is the Bragg angle.

2 $$D = \frac{0.9\lambda }{{\beta \;\cos \theta }}$$

where D is the crystallite size in NPs, (FWHM) K is the Scherrer constant with a value ranging from 0.9. to 1, λ is the wavelength of the X-ray, θ is Bragg's angle in radians, and β is the full width at half the peak's maximum in radians.

The morphology of synthesized AgNPs was investigated using a field emission scanning electron microscope (FE-SEM) (FEI Nova Nano-SEM 450, Suite 1001, New York, NY, USA) with ultra-high resolution at low voltage (10 kV), capturing surface morphology and 100,000 × magnification after gold coating using a Cressington sputter coater (Watford, UK) in the presence of argon gas as reported [30]. Moreover, CP-AgNPs' size and morphology were examined at a magnification of 100,000 × using Transmission Electron Microscopy (TEM-JEOL, USA) with a voltage of 60 kV by dropping few drops of appropriately diluted NPs on copper grid, and then stored in desiccator overnight before observation before analyzing using the instrument. CP-AgNPs were subjected to zeta potential and dynamic light scattering (DLS) experiments employing a Malvern DLS instrument zetasizer. Additionally, the electrochemical characteristics were tested over on a ZIVE MP1 multi-channel electrochemical workstation. Additionally, the quantification of AgNPs was determined by inductively coupled plasma emission spectroscopy (ICP-OES; Avio 500, PerkinElmer, USA).

#### Biological activities

##### Antibacterial and antifungal activity

The antibacterial activity of CPL-AgNPs was studied using a well diffusion method against Gram-positive *(Bacillus subtilis-*MTCC 121 T*, Bacillus megaterium-*NCTC 6094) and Gram-negative (*Shigella flexneri-*MTCC 1457) bacteria*.* Nutrient Agar media was used for the growth of these bacteria, 28 gm of nutrient Agar was added in 1000 mL of distilled water and autoclaved (LSC-05 Labline Stock center Mumbai, Maharashtra, India) for 20 min. The media was poured into sterile petri plates and allowed to solidify. Later, a 100 µL of the active culture of bacteria (10^6^ CFU mL^−1^) was spread over the plate using cotton swabs, and wells were made in each petri dish using stainless steel cork-borer. Then about 100 µL of CPL-AgNPs stock solution was poured into each well separately. Antibiotics such as ampicillin and tetracycline were tested as a control. The plates were transferred to the refrigerator (Godrej, Maharashtra, India) for 30 min until the test samples diffused into media. Subsequently the pates were transferred to an incubator at 37 °C for 24 h and zone of inhibition was measured using an antibiotic zone scale (Himedia, Maharashtra, India). Additionally, the antifungal activity of CPL-AgNPs was studied against the fungi (*Trichoderma viride-*ITCC 1433*, Penicillium crysogenum*-NCPF 2802*,* and *Aspergillus niger*-MTCC 12975) through a well diffusion method. Briefly, 39 gm of potato dextrose Agar was added into 1000 mL of sterile distilled water and poured in sterile petri triplets, after the media solidified, 100 µL of fresh fungus culture was spread using cotton swab end wells were made by using a cork-borer. A 100 µL of AgNPs was poured into each well separately, whereas antibiotics (ampicillin and tetracycline) were used as a control. Plates were kept in a refrigerator (Godrej, Maharashtra, India) for diffusion after that it was transferred to an incubator at 37 °C for 24 h. After the incubation time zone of inhibition was observed and measured using an antibiotic zone scale.

##### Hydrogen peroxide scavenging and reducing power assay

The hydrogen peroxide scavenging activity was studied by dissolving 50 µL of CPL-AgNPs (stock solution) with 5 mM of 10 mL hydrogen peroxide solution followed by incubation for 20 min at room temperature. Ascorbic acid was tested as standard and the prepared solution absorbance was measured at 610 nm using a spectrophotometer (Spectramax M3, Thermo Scientific, Waltham, MA, USA). The percentage of hydrogen peroxide scavenging assay activity was calculated using the Eq. (1).

The reducing power of AgNPs was tested as reported [10]. In brief, 10 mL of AgNP solution was mixed with 2.5 mL of phosphate buffer (200 mM, pH 6.6) and 2.5 mL of potassium fairy cyanide (1%). The mixture was incubated at 50 °C for 20 min and cooled down rapidly then 2.5 mL of TCA (10%) was added in the solution and centrifuged (CM-12plus, Maharashtra, India) at 3000 rpm for 8 min. Whereas BHT was used as positive control and phosphate buffer was used as negative control. The supernatant was collected, and an equal amount of distilled water was added, and 1 mL of ferric chloride (0.1%) was mixed in the solution. The percentage of reducing power was calculated from the optical density measured at 700 nm.

3 $${\text{Activity}}(\% ) = \frac{{\left( {{\text{A}}\;{\text{blank}} - {\text{A}}\;{\text{sample}}} \right)}}{{{\text{A}}\;{\text{blank}}}} \times 100$$

Whereas A_Blank_ absorbance without extract and A_sample_ is absorbance with extract.

##### Anti-inflammatory efficacy through inhibition of albumin denaturation and membrane stabilization

The anti-inflammatory activity of CPL-AgNPs (test) and aspirin (standard) was studied using a previous reported method [31]. The reaction mixture containing an equal quantity of CPL-AgNPs and bovine albumin (1%). The acidic pH of the reaction was maintained using a small quantity of HCl. Later the reaction mixture was incubated at 51 °C for 20 min and, after that the reacting mixture was kept at room temperature for cooling, and absorbance was measured at 660 nm using UV–visible spectrometry. The percentage of inhibition was calculated using Eq. (3).

Furthermore, the efficacy of AgNPs as membrane stabilizer was studied using human blood. In brief, fresh human blood was collected from the pathological lab of the hospital (Nilangekar Hospital, Maharashtra, India) and 10 mL of blood was centrifuged (CM-12plus, Maharashtra, India) at 2000 rpm for 15 min and washed with an isotonic solution thrice for membrane stabilization assay. Later, the measured volume of blood was reconstituted as 10% (v v^−1^) suspension with normal saline. The reaction mixture containing 1 mL of red blood cell (10%) suspension with CPL-AgNPs (test) and aspirin (standard) was incubated over a water bath at 56 °C for 30 min. After cooling the above mixture was centrifuged at 2500 rpm for 5 min. The absorbance of the supernatant was measured using a spectrophotometer at 560 nm and membrane stabilization (%) was calculated by using Eq. (3).

##### Antidiabetic activity

The antidiabetic efficacy of AgNPs was tested using a modified method reported [10]. The reaction mixture containing individual 250 mL of CPL-AgNPs (test) and metformin (commercial drug used in management of diabetes) solution, and 250 µL of 2% starch, were homogeneously mixed and incubated at 20 °C for 3 min. After the incubation of 250 µL, dinitrosalacylic acid was added and kept over a water bath followed by the addition of 250 µL of α-amylase (0.25 mg mL^−1^). Later the mixture was incubated at 37 °C for 15 min and allowed to cool down at room temperature. The absorbance was measured at 540 nm using a spectrophotometer and the α-amylase inhibition efficacy (%) was calculated using Eq. (3).

#### Electrochemical activities

##### Synthesis of silver nanoparticles and copper sulphate electrode

*Preparation of the CPL-AgNPs electrode* AgNPs-based electrode for capitative assessment was fabricated as reported [23]. Briefly, 80 wt (%) CPL-AgNPs powder, 15 wt (%) carbon black, 5 wt (%) polyvinylidene fluoride (PVDF), and N-methyl 2-pyrrolidone (NMP) were used for the slurry preparation. Then, the homogeneous slurry was coated over a flexible stainless-steel substrate (1 × 1 cm^2^ area) and heated at 60 °C for 1 h using a hot air oven. The mass of material loaded on the active electrode was 0.94 mg cm^−2^. The coated electrode was further used as a positive electrode to fabricate an asymmetric solid-state device.

*The preparation of the CuS electrode* The CuS electrode was fabricated as reported [32]. Briefly, 0.1 M CuSO_4_ and 2.5 mL of TEA were combined under stirring conditions. Subsequently, HCl was added to maintain a pH of 3 and 0.1 M sodium thiosulfate was introduced. The resulting solution was then stored in an oven at 70 °C for 2 h. The coated electrode, with a mass loading of 0.93 mg cm^−2^, was later utilized as the negative electrode in the fabrication of an asymmetric solid-state device.

##### Fabrication of device

*Preparation of (PVA)-Na*_*2*_*SO*_*4*_* gel electrolyte* The PVA-Na_2_SO_4_ gel electrolyte was prepared as reported [33]. In brief, 3 gm of PVA was dissolved in 30 mL of DDW by heating at 70 °C using constant stirring. After 4 h of stirring, freshly prepared 1 M Na_2_SO_4_ (10 mL) as electrolyte due to it provision to provide a stable electrochemical environment and effective supercapacitors performance was added slowly to the PVA solution and stirred at room temperature to form a clear and viscous solution. The prepared transparent and viscous PVA-Na_2_SO_4_ gel was used as an electrolyte to fabricate a solid-state asymmetric supercapacitor.

*Fabrication of hybrid solid-state device* The hybrid solid-state device was fabricated as reported [34]. Briefly, CPL-AgNPs electrodes were used as the positive electrode and CuS as the negative electrode for the fabrication of a hybrid asymmetric supercapacitor device. The hybrid asymmetric super-capacitor device CPL-AgNPs//PVA-Na_2_SO_4_//CuS was assembled using a large area (5 × 5 cm^2^) of positive and negative electrodes with PVA-Na_2_SO_4_ gel electrolyte. For the solid-state device fabrication, the electrodes were mass loaded with 0.932 mg cm^−2^, of active material each. At first, the electrodes were soaked with PVA-Na_2_SO_4_ electrolyte, stacked on each other in a sandwich-like format, and pressured under 0.5-ton hydraulic pressure.

#### Calculations for specific capacitance, capacity, specific energy, and specific power density

Based on GCD characteristic curves, specific capacitance (Cs) and capacity (C) were measured using the following equations:

4 $$\text{Cs }= \frac{\text{I}\times \Delta \text{t }}{\Delta \text{V}\times \text{ m}}$$

5 $$\text{Cs }= \frac{\text{I}\times \Delta \text{t}\times \text{A}}{\text{m}}$$

Here *C*_*s*_ represents the specific capacitance (F g^−1^), and *C* is the capacity (C g^−1^) of the prepared material. I is the discharge current density (A cm^−2^, Δ*t* is the discharge time (s), Δ*V* is the potential window (V), *A* is the unit area of the electrode (cm^2^), and *m* represents the mass of the active material (g cm^−2^).

For optimal super-capacitive performance of the fabricated devices, the precise mass ratio between the positive (CPL-AgNPs) and negative electrode (CuS) is necessary and was evaluated by the theory of charge balance (Q^+^ = *Q*^−)^. The mass balance was obtained using the following equation:

6 $$m^{ + } /m^{ - } = c^{ - } \times \Delta v^{ - } /c^{ + } \times \Delta v^{ + }$$

Here *m*^+^ or *m*^−^, *C*^+^ or *C*^−^, and Δ*V*^+^ or Δ*V*^−^ are the mass (g cm^−2^), specific capacitances (F g^−1^), and potential window (V) of the positive and negative electrode, respectively.

The specific energy (Wh kg^−1^) and power (W kg^−1^) of the fabricated hybrid asymmetric supercapacitor devices are also calculated from the GCD characteristic curves using the following equations:

7 $$S.E = \, 0.5 \, \times C_{s} \times (\Delta V)^{2} / \, 3.6$$

8 $$S.P = E \times 3600 \, / \, \Delta t$$

Here, *E* and *P* reflect the specific energy and specific power of the fabricated device, *C*_*s*_ represents specific capacitance, *V* represents the applied potential window, and Δ*t* indicates the discharge time.

## Statistical analysis

All experiments were performed in triplicate and data presented with standard deviations [SD] and error bars.

## Results and discussion

### Phytochemical analysis

It has been reported that *C. procera* is an important ayurvedic plant that has various medicinal uses to treat various diseases in several previous studies [35]. The aqueous extract of CPL was subjected to phytochemical analysis to analyze the presence or absence of phytochemicals that act as reducing agents for the synthesis of AgNPs. The results of the phytochemical analysis demonstrated that the plant leaves contain alkaloids, anthroquione, carbohydrates, diterpine, glycoside, phenol, phlobatanin, and terpenoids (Table S1 and Table S2 and Fig. S1). These results suggest that *C. procera* extract have potential of reducing the metals to metallic nanoparticles. In several study phenolic-rich extract has been claimed for reducing the silver nitrate to AgNPs [2, 10, 13, 16, 18, 19, 36].

### UV–visible spectral analysis

Bio-reduction of silver ions to AgNPs was monitored using the UV visible spectroscopy technique. The optimization of AgNPs synthesis was studied by measuring the absorbance in the scanning range of 300–700 nm. The color change indicated the formation of NPs and it is further confirmed by the characteristic surface plasm resonance (SPR) peak around 400 nm [37]. The color change occurred due to the reaction in which silver ions are converted into AgNPs**.** The spectral results indicated the formation of absorbance of peak around 400 nm. The synthesis of NPs is generally influenced by various optimization parameters, including temperature, reaction time, and extract volume from plant leaves. Using UV–visible spectrometry to track each parameter, the changes that occurred in several significant variables associated with the experiment were examined, including reaction time (4 h, 8 h, 12 h, 16 h, 20 h, and 24 h), volume ratio (1:1, 1:2, 1:3, 1:4, 1:5), (silver nitrate to plant extract volume), and temperature (4 °C, 20 °C, 40 °C, and 60 °C).

#### Effect of silver nitrate concentration

The effect of AgNO_3_ concentration affects the synthesis of AgNPs is presented in Fig S2(A). The physiological reduction of silver ions to AgNPs was observed using UV–Vis spectroscopy where the reduction, synthesis, and optimization of AgNO_3_ to AgNPs in aqueous solutions within the scanning range of 300–700 nm was monitored. Numerous studies revealed that the appearance of a characteristic surface plasmon resonance (SPR) peak of AgNPs at 400 nm resulted in colorless AgNO_3_ changing into various brown color forms [13, 18]. These findings showed that the reduction of AgNO_3_ measured at 5 mM led to the development of a peak at ~ 400 nm. A similar range of 0.5 mM, 1 mM, 2 mM, 4 mM, and 6 mM of silver nitrate concentrations was used to fabricate metallic NPs using *H. abyssinica*. The results demonstrated that at a concentration of 4 mM AgNO_3_ solution, a characteristic SPR peak and maximum absorbance were observed at a wavelength of 406 nm and considered to be the ideal value for the synthesis of AgNPs [38]. Moreover, in another investigation the impact on the synthesis of AgNPs using *V. amygdalina* extract, the concentration of the AgNO_3_ was changed ranging from 1 to 10 mM resulting in an increase in the production of NPs at the concentration of 8 mM. However, the absorption peaks of AgNPs were broader and less strong at low AgNO_3_ concentrations. Whereas the absorbance intensity progressively rises, the SPR peak moves to a longer wavelength direction, making the absorbance peak sharper and more intense. Reduced absorbance results from a further rise in precursor concentration above the threshold, suggesting that the yield of NPs tends to decrease at higher concentrations. An increase in nucleus formation that led to the synthesis of more nanoparticles may be the cause of the increase in SPR peak intensities [39].

#### Effect of temperature

The UV–Vis spectra were recorded for the reaction mixture stored at 4 °C, 20 °C, 40 °C, 60 °C and 80 °C by keeping the other optimal conditions constant. At different temperatures, the color changes from yellowish to dark brown was observed. It was perceived that the maximum temperature recorded at 40 °C with a maximum length of 400 nm shows a higher rate of AgNPs synthesis, which indicates the ability of *C. procera* leaves extract to reduce silver ions (Fig. S2B). A higher temperature causes the molecules' kinetic energy to increase, the consumption of silver ions to occur more quickly, and the probability of particle size expansion to decrease. Consequently, at higher temperatures, smaller particles with a virtually uniform size distribution develop [40].

#### Effect of extract to volume ratio of silver nitrate

The effect of volume ratio on the synthesis of AgNPs was studied by keeping 5 mM AgNO_3_ solution constant and varying the volume of leaves extract (1:1, 1:2, 1:3, 1:4, and 1:5). The results indicated that the maximum optimized condition was recorded at 1:4 volume ratio with a maximum length of 400 nm shows a higher rate of NPs synthesis, which indicates the ability of *C. procera* leaves extract to reduce silver ion (Fig. S2C). Low absorption at 1:1, 1:2, 1:3, and 1:5 indicated a slow or incomplete reduction of silver ions to AgNPs. The quick reduction of Ag + to Ag metal nanoparticles may be attributed to the abundance of phenolic compounds found in plant extract [10]**.**

#### Effect of reaction time

The effect of reaction time was studied by monitoring the reaction of aqueous plant extract and silver nitrate solution for 0 h, 2 h, 4 h, 8 h, and 16 h, at 40 °C. UV–visible measurement was recorded at different periods of incubation. The movement of CPL extract reacts with the silver nitrate solution leading to color change within 8 h (Fig. S2D) where the maximum quantity of AgNPs was synthesized in the solution. The absorption intensity significantly decreases with additional reaction time, suggesting a drop in CPL-AgNPs concentration. This might be due to aggregation forms and, as a result, increased particle size, which settles down and makes it challenging to identify using UV–Vis spectroscopy [41].

### Characterization of silver nanoparticles

#### X-ray diffraction and fourier-transform infrared spectroscopy

**Fig. 2:**
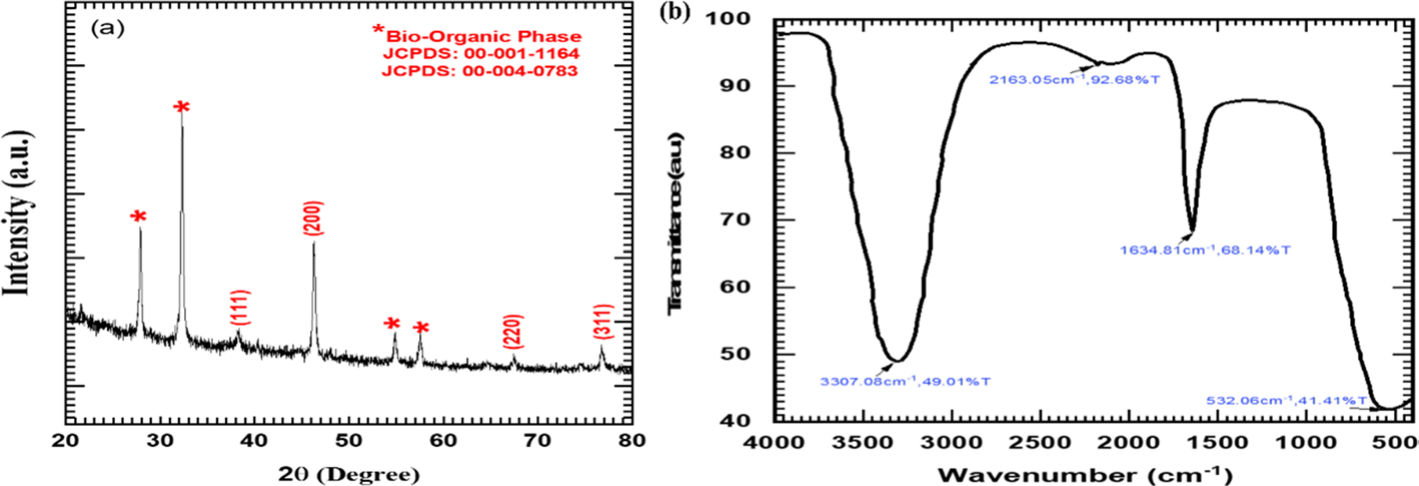
X-ray diffraction pattern of biosynthesized CPL-AgNPs (**a**) and FTIR analysis of CPL-AgNPs (**b**)

The synthesized CPL-AgNPs were characterized using XRD and FTIR. The XRD patterns Fig. 2a of CPL-AgNPs show indexed planes of (111), (200), (220), and (311), indicating a face-centered cubic morphology, with symbol “*” indicating the bio-organic phase. However, the XRD spectra presented additional minor peaks attributed to the presence of phytochemicals crystallites. The crystallite size analysis showed that the AgNPs have an average crystalline size of 36.10 nm, results agreed with obtained TEM particle size of NPs. Moreover, the finding is consistent with the JCPDS files 00-001-1164 and 00-004-0783. FTIR analysis presented in Fig. 2b was used to identify the biomolecules involved in the reduction of silver ions. Intense absorption at ~ 3307 cm^−1^ which is responsible for O–H stretch in alcohols and phenols, ~ 2163 cm^−1^ represents a triple bond of alkynes (C≡C), ~ 1634 cm^−1^ presenting the presence of the C=O stretch in carbonyl groups, often found in ketones, aldehydes, and esters, and ~ 532 cm^−1^ is responsible for metal–oxygen (M–O) vibrations, indicating the presence of a metal–oxygen bond. A previous study suggested that FTIR peak observed between ~ 1634 cm^−1^ and ~ 1350 cm^−1^ attributed to C-H bending of alkane at methyl group usually shift in case of synthesized AgNPs, compared to capping and reducing agent confirm the reduction of AgNO_3_ to its NPs form [13]. The biomolecules present within leaf extract likely act as reducing, capping, and stabilizing agents during the synthesis of AgNPs, as shown broad absorption features in the FTIR spectra [10].

#### Morphology analysis

**Fig. 3:**
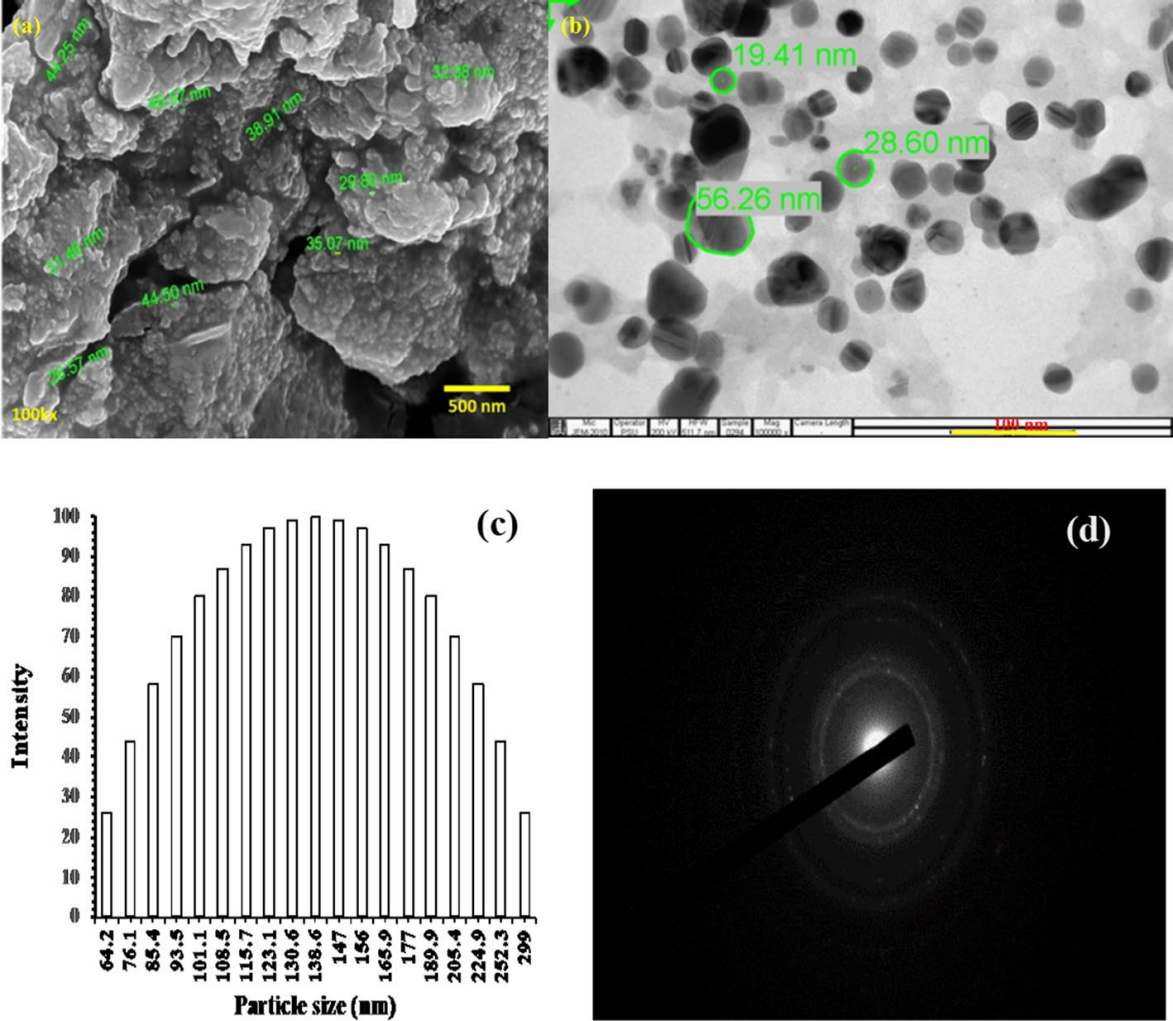
Field emission scanning electron microscopy micrograph analysis of CPL-AgNPs (**a**), transmission electron microscopy pictures at 50,000 X (**b**) displaying the spherical to cubic-shaped CP-AgNPs. Silver nanoparticle particle size histogram obtained through DLS (**c**) and SAID pattern of AgNPs (**d**)

Field emission scanning electron microscopy is widely used when alone scanning electron microscopy characterization of a specific material is unable to yield a clear or acceptable morphology due to its superior resolution [1]. AgNPs are rounded and clumped together as observed from FESEM image (Fig. 3a). FE-SEM analysis of CPL-AgNPs shows the homogeneous spherical-to-cubic shape, a particle size range of 29–46 nm. Additionally, TEM and DLS were used to determine and confer the average diameter, shape, and size morphology of CPL-AgNPs. A TEM picture at 50,000 X revealed that the particles were monodispersed and comparable in size, ranging from 19.41 to 56.26 nm. The DLS measurement was conducted in an aqueous environment, although it indicated median and mean particle diameters of 98.05 nm and 108.0 nm, respectively, with polydispersity (PDI) of 0.239. These values are significantly higher than the size obtained using TEM **(**Fig. 3b and d)**,** since DLS measurement was carried out in an aqueous media, as a result there exist a tendency of measuring the hydration shell of water molecules surrounding the particles **(**Fig. 3c). Moreover, the crystal surface repelling force between CPL-AgNPs exhibiting stabilization by biomolecules led to uniform dispersion with a zeta potential of approximately − 35.1 mV. The formation of CPL-AgNPs with high zeta potential suggests *C. procera* leaf extract exhibit excellent reducing, capping, and stabilizing bioactive compound, which further also confirms the long-term stability of CPL-AgNPs in colloidal form with reduction in chance of possible aggregation among NPs.

### Biological activities

#### Antibacterial and antifungal activity

The antibacterial activity of synthesized CPL-AgNPs was investigated against bacteria such as *B. megabacterium, and B. subtilits.* A prior study showed that AgNPs exhibit a range of antibacterial activity since Gram-positive and Gram-negative bacteria have different cell walls and membranes in terms of composition and structure. Gram-positive bacteria exhibit a strong peptidoglycan coating that acts as a strong barrier that prevents the AgNPs from adhering to or penetrating through [42]. The results for the zone of inhibition of AgNPs against *B. megabacterium*, *B. subtilits*, and *S. flexneri* after the incubation are presented in Table 1 and Fig. **S**3. AgNPs showed efficient anti-bacterial activity, compared to other salts due to their high surface area and charge are believed to aid adhesion to the microbial cell leads to lysis of cells [43]. The electrostatic interaction between positively charged NPs and negatively charges bacterial surface that can induce alteration in the outer membrane integrity of bacteria as well as leakage of cytoplasmic contents, is often adduced as the mechanistic basic of the antimicrobial properties of AgNPs. Similarly, positive ions released by NPs could interact with the negatively charged components (sulphur and phosphorous) of bacterial DNA, and binds with the double-stranded DNA, leading to the disordering of the helical structure by cross-linking within and between the nucleic acids strands. Thus, inducing stress and ultimately bacterial death. However, the fabricated CPL-AgNPs is highly negative charged, hence electrostatic interaction may not hold as the basis of interaction with bacterial cells. It is safer to reason that the proximity of CPL-AgNPs to the bacterial cells in aqueous solution may trigger the release of some secretions to the surrounding milieu that may facilitate the leaching of Ag^+^. Furthermore, the inner and outer membrane of the bacterial cell can be partitioned by their interaction with phenolics, thus rendering them membrane permeable, which may further facilitate the penetration of the released ion from NPs. Hence, single AgNPs interact with DNA and protein effectively, by releasing the silver ion AgNPs which increases the bactericidal activity. In addition, AgNPs generate reactive oxygen space, disrupt on respiratory system, inhibit cell division, and finally lead to cell death [44]**.** Fig. S3 and Table 1 demonstrate the antifungal activity of biosynthesized CPL-AgNPs against fungi *A. niger, P. crysogenum,* and *T. viride*. The results indicated a zone of inhibition was observed around the well containing AgNPs extract whereas no zone was observed around the well that contained only plant leaves extract without nanoparticles. Research revealed that AgNP-induced breakdown of cell membranes increases the log phase of the growth curve, which affects *Candida albicans*' ability to budding. Additionally, free Ag + ions can bind to DNA and sulfur and phosphorus groups in cell membrane components, degrading both substances [45].

**Table 1:** Antibacterial and antifungal activity of biosynthesized CPL-AgNPs

Bacteria	Zone of inhibition (nm)	Fungi	Zone of inhibition (nm)
*B. megabacterium*	18 ± 1.89	*A. niger*	22 ± 2.14
*B. subtilits*	20 ± 1.95	*P. crysogenum*	20 ± 1.98
*S. flexneri*	21 ± 1.99	*T. viride*	18 ± 2.16

#### Antioxidant activity

Primary antioxidants include a vast group of secondary metabolites, especially plant phenols and carotenoids [46–48]. These substances can function as scavengers, hydrogen donors, and single-electron oxygen scavengers due to their high redox potential. According to research, overproduction of hydroxyl radicals, superoxide anions, and hydrogen peroxide can harm DNA cells, living tissues, and their protein and lipid peroxidation processes [49]. The quantity of an antioxidant molecule determines a plant's antioxidant activity on average; plants with more phenolic compounds have higher antioxidant activity. In living organisms, hydrogen peroxide leads to the formation of free radicals which causes severe damage to cells [31]. The hydrogen peroxide scavenging activity of CPL-AgNPs was studied using spectrometer, whereas ascorbic acid was used as a positive control. Inhibition was found to be 29.31 ± 0.21 (%) and 0.13 ± 0.19 (%) for CPL-AgNPs and ascorbic acid, respectively. The results indicated AgNPs exhibit a better scavenging activity, compared with ascorbic acid, due to the structure and characterization of silver nanoparticles. The result confirmed that AgNPs have 4.38% reducing power activity while the standard BHT has 2.08% reducing power activity at very low concentrations due to the presence of phytocompound within the plant extract. The synthesized AgNPs from *A. marmelos* leaves extract have good reducing power activity (20%), compared to the nanoparticles that are synthesized from *C. procera* leaves extract [50].

#### Anti-inflammatory activity

Anti-inflammatory agents are referred to as agents that are responsible for inhibiting protein denaturation. Protein denaturation is the process by which a protein loses its secondary and tertiary structure. This might occur as a result of exposure to specific physical and chemical triggers [51]. Proteins lose their biological functions when denaturation occurs. AgNPs showed anti-inflammatory activity with inhibition of albumin denaturation (%) of 2.08 ± 0.08 (%) which was compared with standard aspirin at 2.54 ± 0.11 (%). Previous studies have reported that the denaturation of protein causes rheumatoid arthritis and inflammation [52].

#### Antidiabetic activity

The suppression of α-amylase and α-glucosidase enzymes is one of the main methods used to treat diabetes. Before glucose is taken into the blood, these enzymes, which are found in saliva, pancreatic juice, and the mucosal brush border of the small intestine, break down carbohydrates and polysaccharides into smaller, more absorbable molecules. In the end, this results in a higher blood glucose concentration. Therefore, inhibiting this two enzymes’ activity essentially lowers the blood's concentration of absorbable glucose, which in turn lowers the postprandial blood glucose level. One of the most successful treatment regimens for diabetes is the inhibition of these two enzymes, α-glucosidase and α-amylase [36]. The results demonstrated that AgNPs exhibit α-amylase inhibitory activity of 83.29 ± 0.26 (%), compared to tested metformin of 2.40 ± 0.18 (%) at tested concentration**.**

### Electrochemical performance

#### Electrochemical capacitive performance of CPL-AgNPs electrodes

**Fig. 4:**
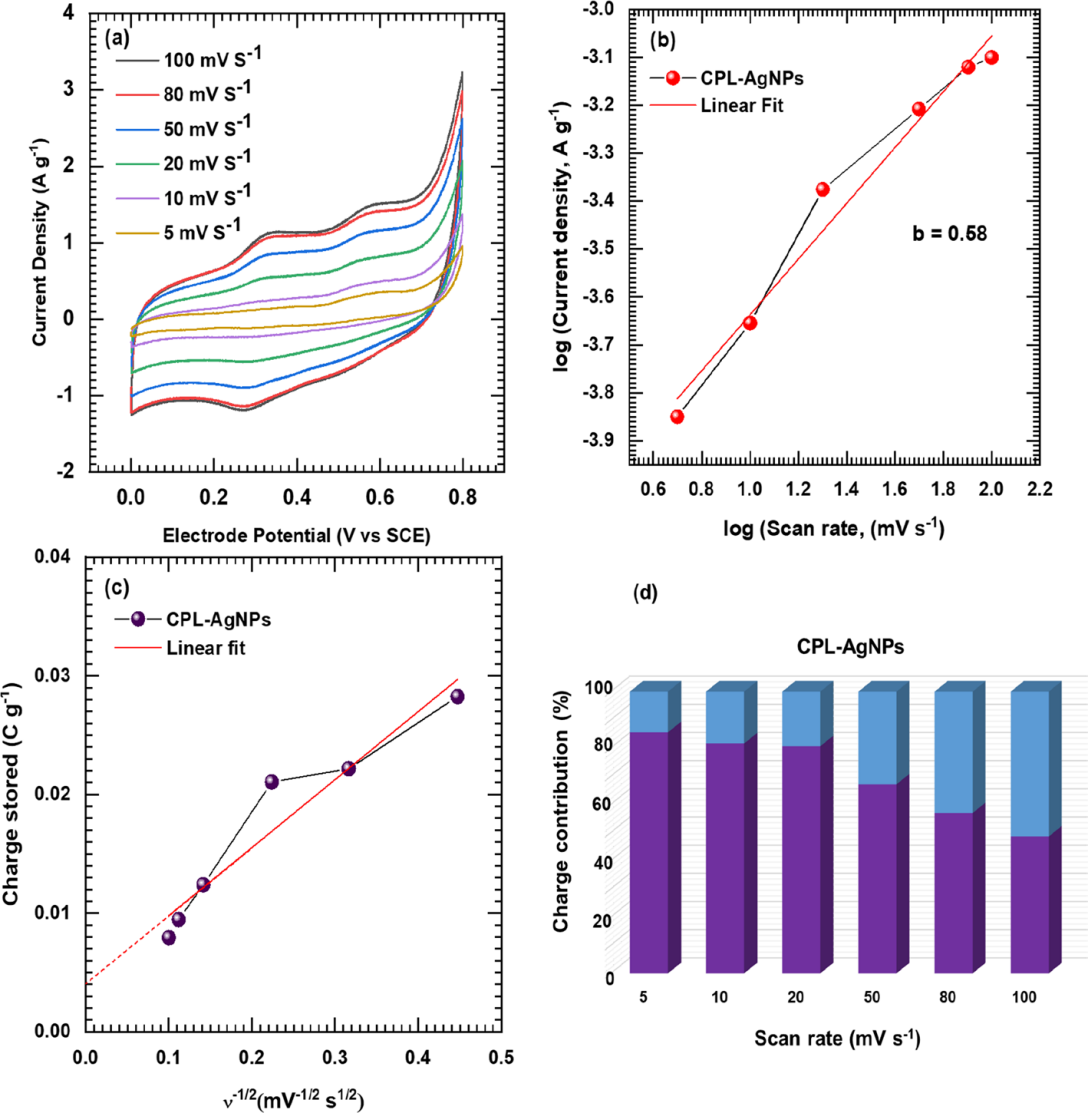
CV curves of CPL-AgNPs electrode at various scan rates from 5 to 100 mV s^−1^ (**a**), calculated contribution of I_surface_ (surface current) and I_bulk_ (bulk current) current density at various scan rates of CPL-AgNPs electrode (**b**), the plot of log (peak current) versus the log (scan rate) for electrode CPL-AgNPs (**c**), and capacitive contribution of CPL-AgNPs from 5 to 100 mV s.^−1^ scan rates (**d**)

The physicochemical properties of the CPL-AgNPs electrode and their impact on super-capacitive performance were analyzed through electrochemical characterization by employing a three-electrode system. This system includes a CPL-AgNPs electrode as the working electrode, a graphite plate as an auxiliary (counter) electrode, and a saturated calomel electrode as the reference electrode. The 1 M Na_2_SO_4_ electrolyte was used to facilitate ion intercalation for charge storage. Figure 4 displays the correlative cyclic voltammetry curves of sample CPL-AgNPs at various scan rates (5, 10, 20, 50, 80, and 100 mV s^−1^) within the potential range of 0–0.8 V SCE.

The CPL-AgNPs electrode exhibits a quasi-rectangular shape with a redox couple, confirming its pseudocapacitive behavior. Charge storage is attributed to a reversible electrochemical reaction in an aqueous Na_2_SO_4_ electrolyte, involving the intercalation and deintercalation of SO_4_^−^ ions during charging and discharging [53]**.** The Cyclic voltammetry analysis indicates an increase in current under the curve maximum with scan rates Fig. 4a. To investigate the storage mechanism, the diffusion control (battery type) and surface capacitive control processes were probed using the power law (Eq. 9),

9 $$I_{p} = a\upsilon^{b}$$

where *I*_p_ represents the current density (peak), υ represents the scan rate, and *a* and *b* are variable factors.

The derived *b* values for the CPL-AgNPs electrode are found to be 0.58 Fig. 4b, confirming the involvement of both diffusion and surface capacitive mechanisms in the charge storage process [21, 22]**.** To further understand the quantitative analysis of the charge storage mechanism, surface capacitive charge, and diffusion-controlled charge (Q_s_—I_surface_ and Q_d_—I_bulk_) in overall volumetric charge response were calculated using the modified power law (Eq. 10),

10 $${\text{Q}}_{{\text{t}}} = {\text{ Q}}_{{\text{s}}} + {\text{ Q}}_{{\text{d}}}$$

The approximate Q_s_ and Q_d_ values were estimated by plotting Q_t_ versus υ^−1/2^ Fig. 4c, and (Eq. 11) measures the charge of both Q_s_ and Q_d_ in total charge contribution,

11 $${\text{Q}}_{{\text{t}}} = {\text{ Q}}_{{\text{s}}} + {\text{ k}}\upsilon^{{ - {1}/{2}}}$$

where Q_s_ can be evaluated from the intercept of the plot Q_t_ vs υ^−1/2^ Fig. 4d, and k is a constant.

The charge contribution diagram for the CPL-AgNPs electrode measured at scan rates of 5–100 mV s^−1^ is provided in Fig. 4d, illustrating that the capacitive contribution (blue color) increases with the scan rate. The electrode exhibits both capacitive and diffusive types of charge storage mechanisms.

**Fig. 5:**
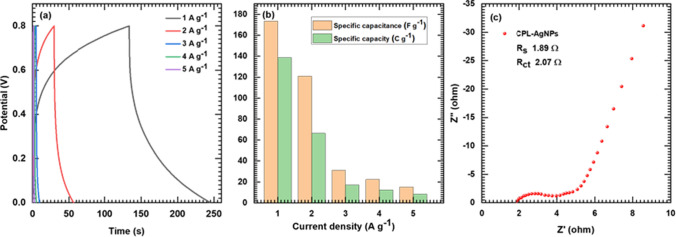
GCD curves of CPL-AgNPs electrode at various current densities from 1 to 5 A g^−1^ (**a**), specific capacity and capacitance of CPL-AgNPs electrode at various current densities (**b**), and Nyquist plot of CPL-AgNPs electrode at OCP (**c**)

The galvanostatic charge–discharge (GCD) curves of the CPL-AgNPs electrode at different current densities (1–5 A g^−1^) and demonstrated in Fig. 5a, The GCD curves exhibit a nontriangular shape, indicating extrinsic pseudocapacitive behavior. The measured specific capacitance and capacity at different current densities are shown in Fig. 5b**.** The CPL-AgNPs electrode demonstrates a maximum specific capacitance (capacity) of 173 F g^−1^ (139 C g^−1^) at a current density of 1 A g^−1^. The CPL-AgNPs electrode exhibits the longest charging-discharging time at 1 A g^−1^ current density, suggesting maximum charge storage capability. Previous results indicate that the preparation of naturally nitrogen-doped carbon nanostructured materials using *Albizia procera* leaves at 850 °C delivered a specific capacitance of 231 F g^−1^, along with charging-discharging cycle stability (97.3% retained after 1000 cycles), confirming the excellent supercapacitance efficiency of this material [23]**.** However, it is important to note that the stainless steel (SS) substrate used in this study does not contribute to the electrochemical reaction to increase capacitance; thus, the capacitance obtained is solely based on the CPL-AgNPs material.

The electrochemical impedance spectroscopy (EIS) analysis was conducted to evaluate the impedance involved in the electrochemical processes of the CPL-AgNPs electrode in the 10–1 MHz frequency range at OCP. The Nyquist plot in Fig. 5c shows the solution resistance (R_s_), and charge transfer resistance (R_ct_). The CPL-AgNPs electrode demonstrates minimum R_s_, R_ct_, (1.89, 2.07 Ω, respectively). The EIS result indicates that the CPL-AgNPs electrode exhibits good capacitive behavior due to a quick charge transport rate (low R_s_ and R_ct_) owing to the preparation of CPL-AgNPs material over the SS substrate. The excellent charge storage ability and low impedance make the electrode a positive electrode (cathode) in hybrid supercapacitor devices.

**Fig. 6:**
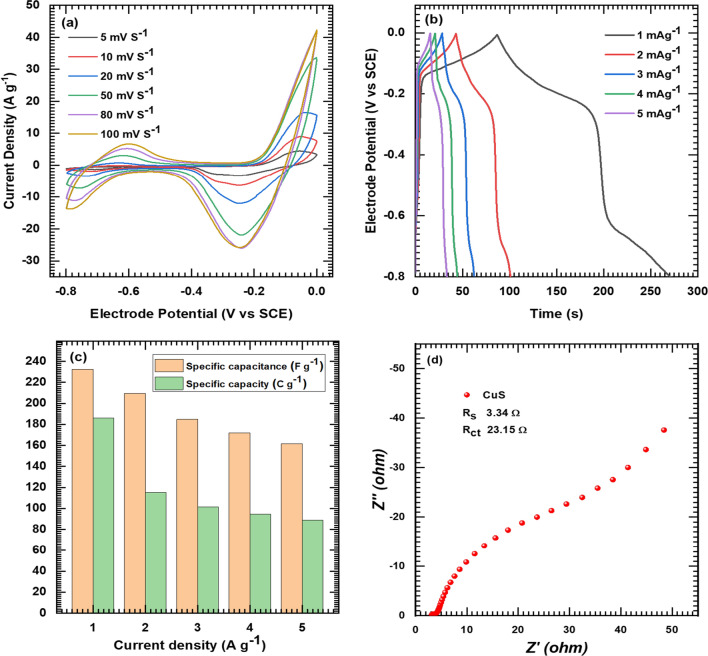
CV curves of CuS electrode at various scan rates from 5 to 100 mV s^−1^ (**a**), GCD curves of CuS electrode at various current densities from 1 to 5 A g^−1^ (**b**), Specific capacity and capacitance of CuS electrode at various current densities (**c**), and Nyquist plot of CuS electrode at OCP (**d**)

The CuS electrode, studied in a 1 M Na_2_SO_4_ solution with a stainless-steel substrate, displayed impressive electrochemical performance. Cyclic voltammetry revealed a notably higher specific capacitance in the CuS electrode. The CV curves demonstrated outstanding high-rate capability at scan rates ranging from 5 to 100 mV s^−1^ Fig. 6a. Galvanostatic charge/discharge measurements confirmed pseudocapacitive behavior, achieving a specific capacitance of 232 F g^−1^ at 1 A g^−1^ and 161 F g^−1^ at 5 A g^−1^ as presented in Fig. 6b and c. The electrode maintained superior rate capability across various current densities. Ragone plot analysis Fig. 6d emphasized its exceptional energy and power density. Electrochemical impedance spectroscopy indicated a larger electro-active surface area and higher electrical conductivity, with solution resistance (R_s_), and charge transfer resistance (R_ct_) values of 3.34 and 23.15 Ω, respectively, further contributing to its overall outstanding performance.

#### Hybrid solid-state supercapacitor device performance

**Fig. 7:**
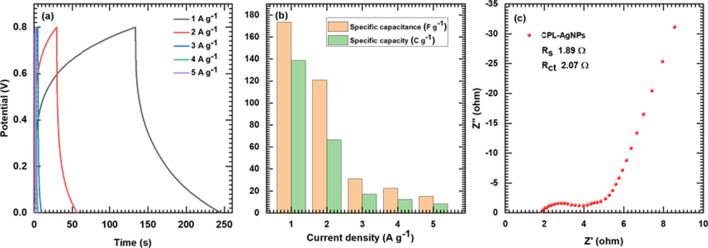
The schematic of the HSSSC device (**a**), a photograph of the fabricated device (**b**)

**Fig. 8:**
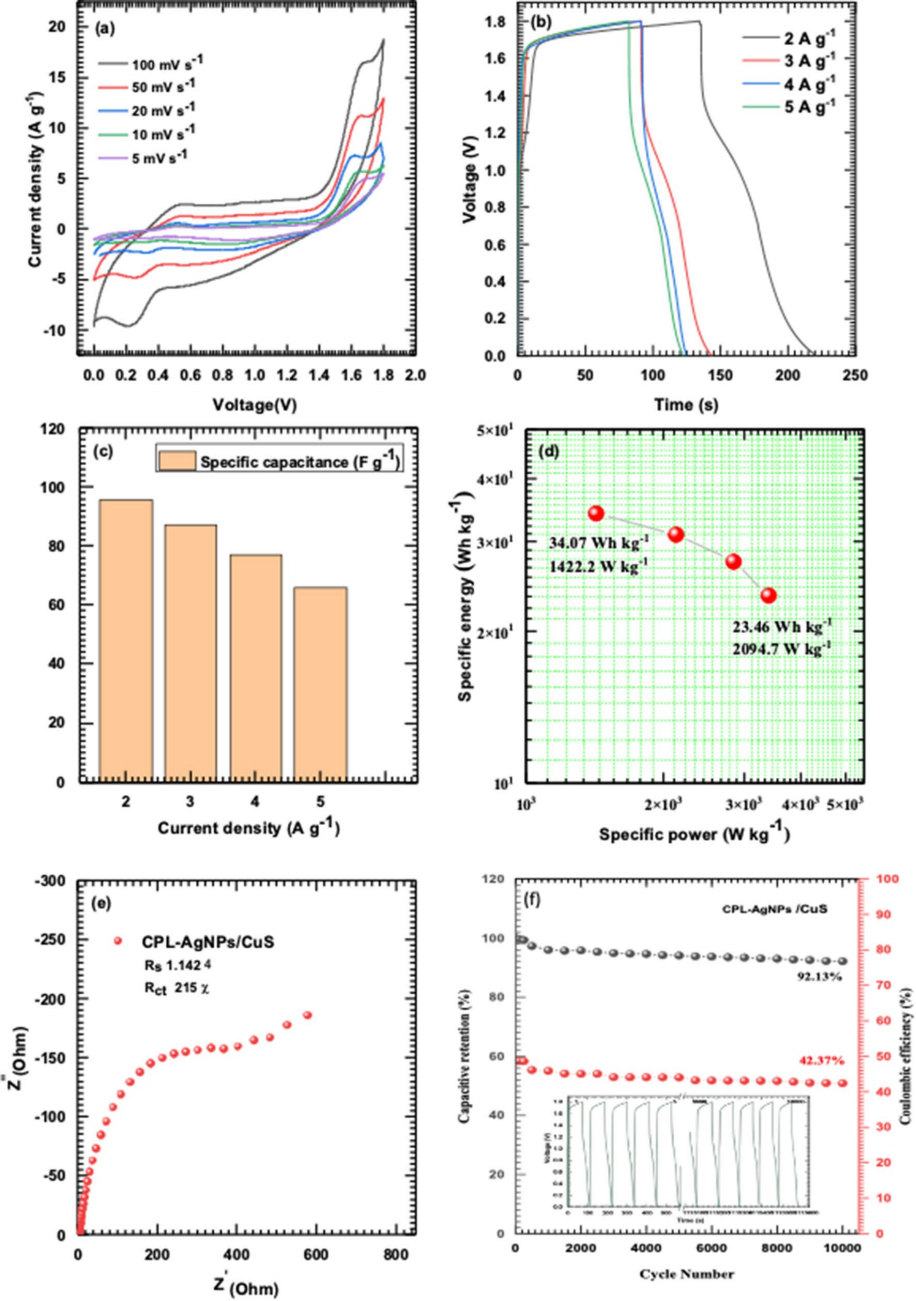
Depicts the CPL-AgNPs/CuS device CV curves at 5–100 mV s^−1^ scan rates (**a**), GCD curves of CPL-AgNPs/CuS electrode at various current densities from 2 to 5 A g^−1^ (**b**), specific capacity and capacitance of CPL-AgNPs/CuS electrode at various current densities (**c**), specific energy vs specific power of CPL-AgNPs/CuS electrode (**d**), nyquist plot of CPL-AgNPs electrode at OCP (**e**), and depicts the capacitive retention and coulombic efficiency of CPL-AgNPs/CuS Device after 10,000 cycles (**f**)

The Hybrid Solid-State Supercapacitor Device Performance (HSSSC) device was fabricated using CPL-AgNPs electrodes and CuS electrodes, with a PVA- Na_2_SO_4_ gel electrolyte serving as a separator to prevent leakage and maintain the device flexibility. Figure 7a illustrates the schematic of the HSSSC device, and photographs of the fabricated device are shown in Fig. 7b. The potential window was varied from 0 to 1.8 V for further HSSSC device study. Figure 8a demonstrates the HSSSC device CV curves at 5–100 mV s^−1^ scan rates, and Fig. 8b shows the GCD curves at distinct 2–5 A g^−1^ current densities. The specific capacitances of the HSSSC device were evaluated from GCD measurements at different current densities and plotted in Fig. 8c, with a maximum specific capacitance of 97 F g^−1^ achieved at a lower current density of 2 A g^−1^ and 63 F g^−1^ at a high 5 A g^−1^ current density.

**Fig. 9:**
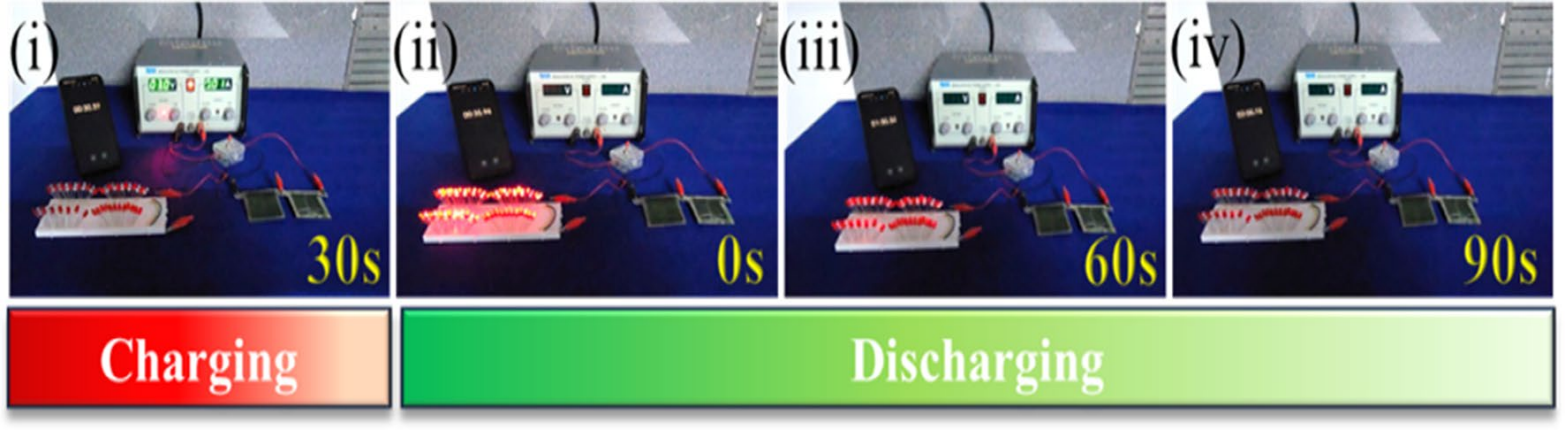
Demonstration of the HSSSC device by glowing 50 red LED (i–iv)

**Fig. 10:**
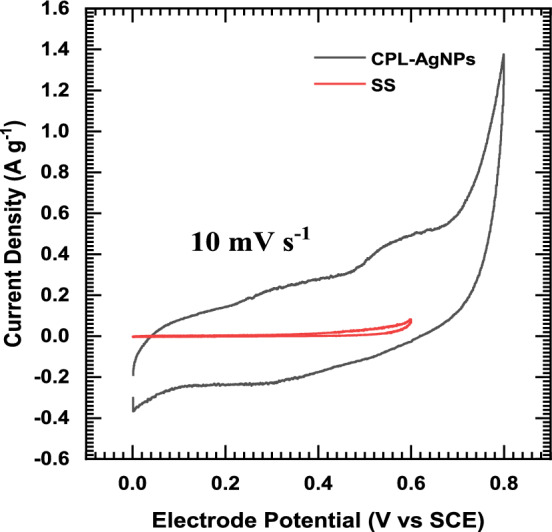
Comparison of electrochemical performance between the current collector and the as-prepared electrode material, demonstrating higher area for the as-prepared electrode material, confirming its superior performance

In this study Fig. 8d displays the HSSSC device Ragone plot, estimating the device's practical potential. The HSSSC device reached 34 Wh kg^−1^ SE at 1.4 kW kg^−1^ SP and maintained up to 23.4 Wh kg^−1^ SE at 2 kW kg^−1^ SP. The R_ct_ value increases 333 Ω, demonstrating decreased conductivity due to surface oxidation. The charge transfer behavior and ion-diffusion properties were evaluated through the EIS measurements after the durability test, and the HSSSC device's Nyquist plots are illustrated in Fig. 8e. The R_S_ value of 1.42 Ω was recorded, indicating good ion diffusion, excellent conductivity, and interaction between the substrate and materials. The capacitive retention and coulombic efficiency are presented in graph Fig. 8f demonstrating that the CPL-AgNPs/CuS device maintains 92.13% of its capacitance over 10,000 cycles whereas, the coulombic efficiency was observed over this period is 42.37%. Furthermore, to examine the practical application of the fabricated device, the energy storage capacity and power output ability were evaluated. The HSSSC device delivers impressive specific energy (34 Wh kg^−1^) and specific power (1.4 kW kg^−1^) against the CPL-AgNPs*-*based hybrid devices, as shown in the Ragone plot Fig. 8d. Two series-connected devices successfully powered up 50 Red light-emitting diodes for 90 s as shown in Fig. 9. This indicates that the device's excellent stability and suitability for long-term energy storage. Moreover, the results of electrochemical performance and energy storage capability of CPL-AgNPs/CuS (Fig. 10) corroborates with the SrMoO_4_ and ZrV_2_O_7_ nanostructures as an electrode material for supercapacitors usage as electrode material for energy storage systems [54, 55].

The study investigates a variety of materials aimed at enhancing supercapacitor performance. Table S3 presents the super-capacitive properties of these materials, including carbon-based functional materials, with their high electrical and thermal conductivity, enabling the development of highly sensitive devices [56]. Flexible electrochromic supercapacitor electrodes using novel transparent conducting substrates, demonstrate great electrochemical performances (13.6 mF cm^−2^, 138.2 F g^−1^) and high coloration efficiency of 80.2 cm^2^ C^−1^ [57]. Silver nanoparticles synthesized through electroless deposition exhibited a maximum specific capacitance of 452 F g^−1^ and specific energy of 27.8 Wh kg^−1^, with impressive cyclic stability, making them suitable for industrial production [58]. Coating redox-active transition-metal oxides like MnO_2_ with conductive Ag nanoparticles led to electrodes with a specific capacitance of 293 F g^−1^, twofold higher than bare MnO_2_, along with high energy/power densities [59]. A cost-effective electroless reduction process anchored Ag nanoparticles onto multi-walled carbon nanotubes, resulting in electrodes with remarkable specific capacitance of 757 F g^−1^ and cyclic stability of 83% over 3000 cycles, offering promising pathways for energy storage applications [60]. Furthermore, the green synthesis of Ag nanoparticles using Kimchi cabbage extract demonstrated effective antibacterial activity and specific capacitance of 424 F g^−1^, presenting an eco-friendly alternative for diverse applications [22]. Additionally, in this work CPL-AgNPs electrode on a flexible SS substrate demonstrated a specific capacitance of 97 F g^−1^, a specific power of 1400 W kg^−1^, an energy density of 34 Wh kg^−1^, and a retention rate of 92%.

These findings underscore the diverse strategies in material synthesis aimed at advancing supercapacitor technology. The benefits of the CPL-AgNPs electrode contributing to excellent electrochemical capacitive performance includes mesoporous and crystalline nature demonstrating a large surface area, minimum electrochemical resistance for rapid charge transfer, crystalline nature facilitating free access to aqueous electrolytic ions, and defect-rich pores providing long-term durability. The CPL-AgNPs electrode displays maximum capacitive performance in terms of high specific power and specific energy with exceptional durability.

## Conclusions

Biological agents can be used to produce nanoparticles at room temperature in an affordable, ecologically friendly approach. Phytochemicals from *Calotropis procera* leaves extracts serve as stabilizing and reducing agents and synthesize CP-AgNPs which are then applied to electrochemical supercapacitors and biomedical applications. Improved biomimetic properties shown by the CP-AgNPs support more research on animals and humans. The small dimensions along with the permeable properties of the CP-AgNPs thin coating, which enable quick and effective ionic transport from electrolyte to the electrode, are responsible for the high capacitive performance. In order to comprehend the fundamental electrochemistry of CP-AgNPs, the study elaborates on their charge storage mechanism. The results that have been presented offer a fresh perspective on a green chemistry strategy for the synthesis of nanoparticles and propose a practical substitute for costly and hazardous conventional methods.

## Supplementary Information

Additional file1 (DOCX 1456 kb)

## Data Availability

Raw data can be made available on request to corresponding authors.

## Abbreviations

AgNO_3_: Silver nitrate
AgNPs: Silver nanoparticles
NPs: Nanoparticles
UV–vis: UV–Visible
XRD: Xray Diffraction
FESEM: Field emission scanning electron microscope
FTIR: Fourier transform infrared spectroscopy
FCC: Face centered cubic
CPL: *Calotropis procera* Leaves
ROS: Reactive oxygen species
SPR: Surface plasma resonance
SS: Stainless steel
SE: Specific Energy
KW: Kilo Watts
Wh: Watts hour
SE: Specific energy
SP: Specific power
CV: Cyclic Voltmeter
CFU: Colony-Forming Unit
pH: Potential of hydrogen
RBC: Red Blood Cell
ZOI: Zone of inhibition
EIS: Electrochemical Impedance Spectroscopy
GCD: Galvanostatic charge/discharge
R_s:_: Solution resistance
R_ct_: Charge transfer resistance
HSSSC: Hybrid solid-state supercapacitor device performance

## References

[1] Kumar A, Jayeoye TJ, Mohite P, Singh S, Rajput T, Munde S, Eze FN, Chidrawar VR, Puri A, Prajapati BG, Parihar A. Sustainable and consumer-centric nanotechnology-based materials: an update on the multifaceted applications, risks and tremendous opportunities. Nano-Struct Nano-Objects. 2024;38: 101148. 10.1016/j.nanoso.2024.101148. 10.1016/j.nanoso.2024.101148

[2] Syukri DM, Singh S, Nwabor OF, Ontong JC, Dejyong K, Sunghan J, Dejyong K, Lethongkam S, Voravuthikunchai SP. Prevention of post-operative bacterial colonization on mice buccal mucosa using biogenic silver nanoparticles-coated nylon sutures. Regenerative Eng Transl Med. 2024. 10.1007/s40883-024-00335-3. 10.1007/s40883-024-00335-3

[3] Syukri DM, Nwabor OF, Singh S, Ontong JC, Wunnoo S, Paosen S, Munah S, Voravuthikunchai SP. Antibacterial-coated silk surgical sutures by ex situ deposition of silver nanoparticles synthesized with Eucalyptus camaldulensis eradicates infections. J Microbiol Methods. 2020;174: 105955. 10.1016/j.mimet.2020.105955. 32442657 10.1016/j.mimet.2020.105955

[4] Jayeoye TJ, Nwude EF, Singh S, Prajapati BG, Kapoor DU, Muangsin N. Sustainable synthesis of gold nanoparticles for drug delivery and cosmeceutical applications: a review. BioNanoScience. 2024. 10.1007/s12668-024-01436-7. 10.1007/s12668-024-01436-7

[5] Kumar A, Shah SR, Jayeoye TJ, Kumar A, Parihar A, Prajapati B, Singh S, Kapoor DU. Biogenic metallic nanoparticles: biomedical, analytical, food preservation, and applications in other consumable products. Front Nanotechnol. 2023. 10.3389/fnano.2023.1175149. 10.3389/fnano.2023.1175149

[6] Singh SA, Vellapandian C, Shah DD, Jayeoye TJ, Chorawala MR, Singh S, Prajapati BG. Valorised calcium-rich biomass from fish waste and eggshells in the fabrication of antibacterial scaffold for wound healing applications: a review. Waste Biomass Valorization. 2024;15(4):1917–41. 10.1007/s12649-023-02302-5. 10.1007/s12649-023-02302-5

[7] Begum RF, Singh S, Prajapati B, Sumithra M, Patel RJ. Advanced targeted drug delivery of bioactive agents fortified with graft chitosan in management of cancer: a review. Curr Med Chem. 2024. 10.2174/0109298673285334240112104709. 38415441 10.2174/0109298673285334240112104709

[8] Mohite P, Shah SR, Singh S, Rajput T, Munde S, Ade N, Prajapati BG, Paliwal H, Mori DD, Dudhrejiya AV. Chitosan and chito-oligosaccharide: a versatile biopolymer with endless grafting possibilities for multifarious applications. Front Bioeng Biotechnol. 2023. 10.3389/fbioe.2023.1190879. 37274159 10.3389/fbioe.2023.1190879 PMC10235636

[9] Ahmed B, Bilal Tahir M, Sagir M, Hassan M. Bio-inspired sustainable synthesis of silver nanoparticles as next generation of nanoproduct in antimicrobial and catalytic applications. Mater Sci Eng B. 2024;301: 117165. 10.1016/j.mseb.2023.117165. 10.1016/j.mseb.2023.117165

[10] Nagime PV, Singh S, Shaikh NM, Gomare KS, Chitme H, Abdel-Wahab BA, Alqahtany YS, Khateeb MM, Habeeb MS, Bakir MB. Biogenic fabrication of silver nanoparticles using calotropis procera flower extract with enhanced biomimetics attributes. Materials. 2023;16(11):4058. 37297192 10.3390/ma16114058 PMC10254777

[11] Jayeoye TJ, Singh S, Eze FN, Olatunji OJ, Olatunde OO, Omaka ON, Odogiyon OB, Okpara KE. Exploration of biocompatible ascorbic acid reduced and stabilized gold nanoparticles, as sensitive and selective detection nanoplatform for silver ion in solution. Plasmonics. 2024. 10.1007/s11468-024-02413-2. 10.1007/s11468-024-02413-2

[12] Jayeoye TJ, Eze FN, Singh S, Olatunde OO, Benjakul S, Rujiralai T. Synthesis of gold nanoparticles/polyaniline boronic acid/sodium alginate aqueous nanocomposite based on chemical oxidative polymerization for biological applications. Int J Biol Macromol. 2021;179:196–205. 10.1016/j.ijbiomac.2021.02.199. 33675826 10.1016/j.ijbiomac.2021.02.199

[13] Singh S, Nwabor OF, Sukri DM, Wunnoo S, Dumjun K, Lethongkam S, Kusolphat P, Hemtanon N, Klinprathum K, Sunghan J, Dejyong K, Lertwittayanon K, Pisuchpen S, Voravuthikunchai SP. Poly (vinyl alcohol) copolymerized with xanthan gum/hypromellose/sodium carboxymethyl cellulose dermal dressings functionalized with biogenic nanostructured materials for antibacterial and wound healing application. Int J Biol Macromol. 2022;216:235–50. 10.1016/j.ijbiomac.2022.06.172. 35780920 10.1016/j.ijbiomac.2022.06.172

[14] Puri A, Mohite P, Patil S, Chidrawar VR, Ushir YV, Dodiya R, Singh S. Facile green synthesis and characterization of Terminalia arjuna bark phenolic–selenium nanogel: a biocompatible and green nano-biomaterial for multifaceted biological applications. Front Chem. 2023. 10.3389/fchem.2023.1273360. 37810585 10.3389/fchem.2023.1273360 PMC10556707

[15] Kaushal A, Khurana I, Yadav P, Allawadhi P, Banothu AK, Neeradi D, Thalugula S, Barani PJ, Naik RR, Navik U, Bharani KK, Khurana A. Advances in therapeutic applications of silver nanoparticles. Chem Biol Interact. 2023;382: 110590. 10.1016/j.cbi.2023.110590. 37268200 10.1016/j.cbi.2023.110590

[16] Singh S, Chunglok W, Nwabor OF, Ushir YV, Singh S, Panpipat W. Hydrophilic biopolymer matrix antibacterial peel-off facial mask functionalized with biogenic nanostructured material for cosmeceutical applications. J Polym Environ. 2022;30(3):938–53. 10.1007/s10924-021-02249-5. 10.1007/s10924-021-02249-5

[17] Vyas J, Raytthatha N, Singh S, Prajapati BG, Mohite P, Munde S. Sustainable sources of raw materials as substituting biomaterials for additive manufacturing of dental implants: a review. Periodontal Implant Res. 2024;8(1):3. 10.1007/s41894-024-00130-x. 10.1007/s41894-024-00130-x

[18] Ontong JC, Singh S, Nwabor OF, Chusri S, Voravuthikunchai SP. Potential of antimicrobial topical gel with synthesized biogenic silver nanoparticle using Rhodomyrtus tomentosa leaf extract and silk sericin. Biotech Lett. 2020;42(12):2653–64. 10.1007/s10529-020-02971-5. 10.1007/s10529-020-02971-5 32683522

[19] Syukri DM, Nwabor OF, Singh S, Voravuthikunchai SP. Antibacterial functionalization of nylon monofilament surgical sutures through in situ deposition of biogenic silver nanoparticles. Surf Coat Technol. 2021;413: 127090. 10.1016/j.surfcoat.2021.127090. 10.1016/j.surfcoat.2021.127090

[20] Lee CS, Yoo JE, Shin K, Park CO, Bae J. Carbon nanotube–silver nanowire composite networks on flexible substrates: high reliability and application for supercapacitor electrodes. Phys Status Solidi (a). 2014;211(12):2890–7. 10.1002/pssa.201431538. 10.1002/pssa.201431538

[21] Dhibar S, Das CK. Electrochemical performances of silver nanoparticles decorated polyaniline/graphene nanocomposite in different electrolytes. J Alloy Compd. 2015;653:486–97. 10.1016/j.jallcom.2015.08.158. 10.1016/j.jallcom.2015.08.158

[22] Lokhande AC, Babar PT, Karade VC, Jang JS, Lokhande VC, Lee DJ, Kim IC, Patole SP, Qattan IA, Lokhande CD, Kim JH. A viable green route to produce Ag nanoparticles for antibacterial and electrochemical supercapacitor applications. Mater Today Chem. 2019;14: 100181. 10.1016/j.mtchem.2019.07.003. 10.1016/j.mtchem.2019.07.003

[23] Salve M, Mandal A, Amreen K, Pattnaik PK, Goel S. Greenly synthesized silver nanoparticles for supercapacitor and electrochemical sensing applications in a 3D printed microfluidic platform. Microchem J. 2020;157: 104973. 10.1016/j.microc.2020.104973. 10.1016/j.microc.2020.104973

[24] Khayyat SA, Abaker M, Umar A, Alkattan MO, Alharbi ND, Baskoutas S. Synthesis and characterizations of Cd-doped ZnO multipods for environmental remediation application. J Nanosci Nanotechnol. 2012;12(11):8453–8. 23421230 10.1166/jnn.2012.6801

[25] Alex J, Rajkumar S, PrincyMerlin J, Aravind A, Sajan D, Praveen CS. Single step auto-igniting combustion technique grown CeO2 and Ni-doped CeO2 nanostructures for multifunctional applications. J Alloy Compd. 2021;882: 160409. 10.1016/j.jallcom.2021.160409. 10.1016/j.jallcom.2021.160409

[26] Brozek CK, Zhou D, Liu H, Li X, Kittilstved KR, Gamelin DR. Soluble supercapacitors: large and reversible charge storage in colloidal iron-doped ZnO nanocrystals. Nano Lett. 2018;18(5):3297–302. 10.1021/acs.nanolett.8b01264. 29693400 10.1021/acs.nanolett.8b01264

[27] Angelin MD, Rajkumar S, Ravichandran AT, Merlin JP. Systematic investigation on the electrochemical performance of Cd-doped ZnO as electrode material for energy storage devices. J Phys Chem Solids. 2022;161: 110486. 10.1016/j.jpcs.2021.110486. 10.1016/j.jpcs.2021.110486

[28] Kaur A, Batish DR, Kaur S, Chauhan BS. An overview of the characteristics and potential of calotropis procera from botanical, ecological, and economic perspectives. Front Plant Sci. 2021. 10.3389/fpls.2021.690806. 34220914 10.3389/fpls.2021.690806 PMC8248367

[29] Sivapalan S, Dharmalingam S, Venkatesan V, Angappan M, Ashokkumar V. Phytochemical analysis, anti-inflammatory, antioxidant activity of Calotropis gigantea and its therapeutic applications. J Ethnopharmacol. 2023;303: 115963. 10.1016/j.jep.2022.115963. 36442758 10.1016/j.jep.2022.115963

[30] Singh S, Supaweera N, Nwabor OF, Chaichompoo W, Suksamrarn A, Chittasupho C, Chunglok W. Poly (vinyl alcohol)-gelatin-sericin copolymerized film fortified with vesicle-entrapped demethoxycurcumin/bisdemethoxycurcumin for improved stability, antibacterial, anti-inflammatory, and skin tissue regeneration. Int J Biol Macromol. 2024;258: 129071. 10.1016/j.ijbiomac.2023.129071. 38159707 10.1016/j.ijbiomac.2023.129071

[31] Singh S, Chidrawar VR, Hermawan D, Nwabor OF, Olatunde OO, Jayeoye TJ, Samee W, Ontong JC, Chittasupho C. Solvent-assisted dechlorophyllization of Psidium guajava leaf extract: Effects on the polyphenol content, cytocompatibility, antibacterial, anti-inflammatory, and anticancer activities. S Afr J Bot. 2023;158:166–79. 10.1016/j.sajb.2023.04.029. 10.1016/j.sajb.2023.04.029

[32] Huang K-J, Zhang J-Z, Fan Y. One-step solvothermal synthesis of different morphologies CuS nanosheets compared as supercapacitor electrode materials. J Alloy Compd. 2015;625:158–63. 10.1016/j.jallcom.2014.11.137

[33] Fadakar Z, Nasirizadeh N, Bidoki SM, Shekari Z, Mottaghitalab V. Fabrication of a supercapacitor with a PVA–KOH–KI electrolyte and nanosilver flexible electrodes. Microelectron Eng. 2015;140:29–32. 10.1016/j.mee.2015.05.004. 10.1016/j.mee.2015.05.004

[34] Zou Z, Zhou W, Zhang Y, Yu H, Hu C, Xiao W. High-performance flexible all-solid-state supercapacitor constructed by free-standing cellulose/reduced graphene oxide/silver nanoparticles composite film. Chem Eng J. 2019;357:45–55. 10.1016/j.cej.2018.09.143. 10.1016/j.cej.2018.09.143

[35] Qura Tulain M, Yasin T, Raheem MA. Comprehensive exploration of the pharmacological importance of calotropis (AKK) species. A Review. 2024;8:49.

[36] Jayeoye TJ, Eze FN, Olatunde OO, Singh S, Zuo J, Olatunji OJ. Multifarious biological applications and toxic Hg2+ sensing potentiality of biogenic silver nanoparticles based on securidaca inappendiculata hassk stem extract. Int J Nanomed. 2021;16:7557. 10.2147/IJN.S325996 PMC8597655 34803379

[37] Nwabor OF, Singh S, Ontong JC, Vongkamjan K, Voravuthikunchai SP. Valorization of wastepaper through antimicrobial functionalization with biogenic silver nanoparticles, a sustainable packaging composite. Waste Biomass Valorization. 2021;12(6):3287–301. 10.1007/s12649-020-01237-5. 10.1007/s12649-020-01237-5

[38] Murthy HA, Zeleke TD, Ravikumar C, Kumar MA, Nagaswarupa H. Electrochemical properties of biogenic silver nanoparticles synthesized using Hagenia abyssinica (Brace) JF. Gmel. medicinal plant leaf extract. Mater Res Express. 2020;7(5):055016. 10.1088/2053-1591/ab9252

[39] Tesfaye M, Gonfa Y, Tadesse G, Temesgen T, Periyasamy S. Green synthesis of silver nanoparticles using Vernonia amygdalina plant extract and its antimicrobial activities. Heliyon. 2023;9(6): e17356. 10.1016/j.heliyon.2023.e17356. 37383214 10.1016/j.heliyon.2023.e17356 PMC10293723

[40] Rameshkumar K, Ananthi V, Arun A, Prema P, Veeramanikandan V, Nguyen V-H, Balaji P. Trianthema portulacastrum leaf extract mediated synthesis of silver nanoparticles and elucidation of their larvicidal and antibacterial activities. Mater Today Commun. 2023;35: 105980. 10.1016/j.mtcomm.2023.105980. 10.1016/j.mtcomm.2023.105980

[41] Sakthi Devi R, Girigoswami A, Siddharth M, Girigoswami K. Applications of gold and silver nanoparticles in theranostics. Appl Biochem Biotechnol. 2022;194(9):4187–219. 10.1007/s12010-022-03963-z. 35551613 10.1007/s12010-022-03963-z PMC9099041

[42] More PR, Pandit S, Filippis AD, Franci G, Mijakovic I, Galdiero M. Silver nanoparticles: bactericidal and mechanistic approach against drug resistant pathogens. Microorganisms. 2023;11(2):369. 36838334 10.3390/microorganisms11020369 PMC9961011

[43] Anees Ahmad S, Sachi Das S, Khatoon A, Tahir Ansari M, Afzal M, Saquib Hasnain M, Kumar Nayak A. Bactericidal activity of silver nanoparticles: a mechanistic review. Mater Sci Energy Technol. 2020;3:756–69. 10.1016/j.mset.2020.09.002. 10.1016/j.mset.2020.09.002

[44] Elemike EE, Onwudiwe DC, Arijeh O, Nwankwo HU. Plant-mediated biosynthesis of silver nanoparticles by leaf extracts of Lasienthra africanum and a study of the influence of kinetic parameters. Bull Mater Sci. 2017;40(1):129–37. 10.1007/s12034-017-1362-8. 10.1007/s12034-017-1362-8

[45] Sánchez-López P, Hernández-Hernández KA, Fuentes Moyado S, RnD CN, Smolentseva E. Antimicrobial and virus adsorption properties of Y-Zeolite exchanged with silver and zinc cations. ACS Omega. 2024;9:7554. 38405448 10.1021/acsomega.3c06462 PMC10882595

[46] Chidrawar VR, Singh S, Jayeoye TJ, Dodiya R, Samee W, Chittasupho C. Porous swellable hypromellose composite fortified with eucalyptus camaldulensis leaf hydrophobic/hydrophilic phenolic-rich extract to mitigate dermal wound infections. J Polym Environ. 2023;31(9):3841–56. 10.1007/s10924-023-02860-8. 10.1007/s10924-023-02860-8

[47] Singh S, Chidrawar VR, Hermawan D, Dodiya R, Samee W, Ontong JC, Ushir YV, Prajapati BG, Chittasupho C. Hypromellose highly swellable composite fortified with psidium guajava leaf phenolic-rich extract for antioxidative, antibacterial, anti-inflammatory, anti-melanogenesis, and hemostasis applications. J Polym Environ. 2023. 10.1007/s10924-023-02819-9. 10.1007/s10924-023-02819-9

[48] Singh S, Chunglok W, Nwabor OF, Chulrik W, Jansakun C, Bhoopong P. porous biodegradable sodium alginate composite fortified with Hibiscus Sabdariffa L. calyx extract for the multifarious biological applications and extension of climacteric fruit Shelf-Life. J Polym Environ. 2023;31(3):922–38. 10.1007/s10924-022-02596-x. 10.1007/s10924-022-02596-x

[49] Yoshikawa T, You F. Oxidative Stress and Bio-Regulation. Int J Mol Sci. 2024;25(6):3360. 38542335 10.3390/ijms25063360 PMC10970561

[50] Rama P, Mariselvi P, Sundaram R, Muthu K. Eco-friendly green synthesis of silver nanoparticles from Aegle marmelos leaf extract and their antimicrobial, antioxidant, anticancer and photocatalytic degradation activity. Heliyon. 2023. 10.1016/j.heliyon.2023.e16277. 37255978 10.1016/j.heliyon.2023.e16277 PMC10225894

[51] Lei M, Wang L, Olatunde OO, Singh S, Ovatlarnporn C, Basit A, Olatunji OJ. UPLC–ESI–QTOF–MS profiling, antioxidant, antidiabetic, antibacterial, anti-inflammatory, antiproliferative activities and in silico molecular docking analysis of Barleria strigosa. Chem Biol Technol Agricul. 2023;10(1):73. 10.1186/s40538-023-00451-2. 10.1186/s40538-023-00451-2

[52] Vidya Sagar PSR, Ramadevi D, Basavaiah K, Botsa SM. Green synthesis of silver nanoparticles using aqueous leaf extract of Saussurea obvallata for efficient catalytic reduction of nitrophenol, antioxidant, and antibacterial activity. Water Sci Eng. 2023. 10.1016/j.wse.2023.09.004. 10.1016/j.wse.2023.09.004

[53] Senthilkumar ST, Park J-S, Marcilla R, Palma J, Kim Y. Using redox electrolytes to extend the charge storage capacity in an aqueous hybrid ion battery. Chem Eng J. 2021;411: 128416. 10.1016/j.cej.2021.128416. 10.1016/j.cej.2021.128416

[54] Sathiyan A, Rajkumar S, Dhineshkumar S, Princy Merlin J. Electrochemical performance of SrMoO4 as electrode material for energy storage systems. J Ind Eng Chem. 2024;129:521–30. 10.1016/j.jiec.2023.09.011. 10.1016/j.jiec.2023.09.011

[55] Rajkumar S, Gowri S, Princy Merlin J. Facile fabrication of ZrV2O7 nanostructures as an electrode material for supercapacitors. Inorg Chem Commun. 2023;153: 110896. 10.1016/j.inoche.2023.110896. 10.1016/j.inoche.2023.110896

[56] Shaikh SB, Waikar MR, Mohite RA, Jadhav SB, Lokhande CD, Pawaskar PN. Carbon-based functional materials for optical sensors. In: Sonker RK, Singh K, Sonkawade R, editors. Advanced functional materials for optical and hazardous sensing progress in optical science and photonics. Singapore: Springer; 2023.

[57] Shen L, Du L, Tan S, Zang Z, Zhao C, Mai W. Flexible electrochromic supercapacitor hybrid electrodes based on tungsten oxide films and silver nanowires. Chem Commun. 2016;52(37):6296–9. 10.1039/C6CC01139J. 10.1039/C6CC01139J 27087032

[58] Pandit B, Devika VS, Sankapal BR. Electroless-deposited Ag nanoparticles for highly stable energy-efficient electrochemical supercapacitor. J Alloy Compd. 2017;726:1295–303. 10.1016/j.jallcom.2017.08.068. 10.1016/j.jallcom.2017.08.068

[59] Xia H, Hong C, Shi X, Li B, Yuan G, Yao Q, Xie J. Hierarchical heterostructures of Ag nanoparticles decorated MnO2 nanowires as promising electrodes for supercapacitors. J Mater Chem A. 2015;3(3):1216–21. 10.1039/C4TA05568C. 10.1039/C4TA05568C

[60] Pandit B, Sankapal BR. Highly conductive energy efficient electroless anchored silver nanoparticles on MWCNTs as a supercapacitive electrode. New J Chem. 2017;41(19):10808–14. 10.1039/C7NJ01792H. 10.1039/C7NJ01792H

