# Supplementary material for: Facile synthesis of silver nanoparticles using Calotropis procera leaves: unraveling biological and electrochemical potentials
Source: Discov Nano. 2024 Sep 3;19(1):139. doi: 10.1186/s11671-024-04090-w (PMC11371983; doi:10.1186/s11671-024-04090-w)
Supplement: Supplementary file 1 — Additional file1 (DOCX 1456 kb) [file 11671_2024_4090_MOESM1_ESM.docx]

Facile synthesis of silver nanoparticles using *Calotropis procera* leaves: Unraveling Biological and electrochemical potentials

Pooja V Nagime^1^* • Nishat M Shaikh^2^ • Sohel B Shaikh^3^ • Chandrakant D Lokhande^3^ • Vinod V Patil^4^ • Sheeba Shafi^5^ • Dwi Marlina Syukri^6^ • Vijay R Chidrawar^7^ • Ashwini Kumar^8^, Sudarshan Singh^9,10^*

^1^ Centre of Excellence in Innovative Biotechnology for Sustainable Utilization of Bioresources, Faculty of Agro-Industry, Prince of Songkla University, Hat Yai 90110, Thailand

^2^ Department of Biotechnology, Dayanand Science College, Latur 413512, India

^3^ Department of Medical Physics, Centre for Interdisciplinary Research, D. Y. Patil Education Society, Deemed to be University, Kolhapur 416006, India

^4^ School of Chemical Sciences, Punyashlok Ahilyadevi Holkar, Solapur University, Solapur 413255, India

^5^ Department of Nursing, College of Applied Medical Sciences, King Faisal University, Al-Ahsa 31982, Saudi Arabia

^6^ Faculty of Medicine, Malahayati University, Bandar Lampung, Lampung, 35153, Indonesia

^7^ School of Pharmacy and Technology Management, SVKM’s Narsee Monjee Institute of Management Studies (NMIMS), Deemed-to-university, Jadcharla, Telangana 509301, India

^8^ Department of Mechanical Engineering, School of Engineering and Technology, Manav Rachna International Institute of Research and Studies, Faridabad 121003, Haryana, India

^9^ Office of Research Administration, Chiang Mai University, Chiang Mai 50200, Thailand

^10^ Faculty of Pharmacy, Chiang Mai University, Chiang Mai 50200, Thailand

*Corresponding Authors

Pooja V Nagime ([poojanagime1010@gmail.com](mailto:poojanagime1010@gmail.com))

Sudarshan Singh ([sudarshansingh83@hotmail.com](mailto:sudarshansingh83@hotmail.com))

**Table S1** Phytochemical analysis of CPL extract

| **Phytocompound** | **Reagent** | **Test** | **Interference** |
| --- | --- | --- | --- |
| Alkaloid | Wangers reagent | To the 3 mL of leaves extract few drops of wanger reagent were added | Formation of brown precipitation |
| Anthocyanin | NaOH reagent | To the 2 mL of extract, 2 mL of 1N NaOH was added | Formation of pink, violet, blue, green color |
| Anthroquion | Borntrager’s reagent | To the extract few drops of sulfuric acid, chloroform, benzene, and ammonia were added | Formation of pink, and red color |
| Diterpine | Copper acetate | To the extract, 10 drops of copper acetate were added | Formation of emerald green color |
| Flavonoid | Alkaline reagent | To the extract 2 ml of 0.2% NaOH and 2 drops of diluted acid were added | Formation of colorless solution |
| Glycoside | Sulphuric acid reagent | To the 1 ml of extract 1ml of sulfuric acid was added | Formation of reddish color |
| Phenol | Ferric chloride reagent | To the extract dropwise ferric chloride was added | Formation of red, blue, green, and purple colors |
| Phlobatanin | Hydrochloric acid reagent | To the 2 mL of extract diluted HCl was added | Formation of red precipitation |
| Protein | Xanthproteic reagent | To the 1 mL of extract 1ml of 40% NaOH was added | Formation of yellow color |
| Tannin | Braymer’s reagent | To the 1 mL of extract, two drops of 50% ferric chloride were added | Formation of green color |
| Terpenoid | Salkowaski reagent | To the 5 mL of extract 2mL of chloroform 3mL of sulphuric acid was added | Formation of reddish color |

**Table S2** Results of phytochemical analysis of CPL

| **Sr No.** | **Phytocompound** | **Reagent** | **Result** |
| --- | --- | --- | --- |
| 1 | Alkaloid | Wangers reagent | + |
| 2 | Anthocyanin | NaOH reagent | - |
| 3 | Anthroquione | Brontragers reagent | + |
| 4 | Carbohydrates | Molish test | + |
| 5 | Diterpine | Copper acetate | + |
| 6 | Flavonoid | Alkaline reagent | - |
| 7 | Glycoside | Sulphuric acid regent | + |
| 8 | Phenol | Ferric chloride reagent | + |
| 9 | Phlobatanin | Hydrochloric acid reagent | + |
| 10 | Protein | Xanthproteic reagent | - |
| 11 | Tannin | Braymers reagent | - |
| 12 | Terpenoids | Salkowaski reagent | + |

**Table S3** Super capacitive properties of devices reported in the previous literature.

| Electrode material | Electrolyte | SC (F/g) | Specific energy (Wh/kg) | Specific power (kW/kg) | References |
| --- | --- | --- | --- | --- | --- |
| Ag-composite | 1M H_2_SO_4_ | 138.2 | - | - | [1] |
| Ag/MnO_2_ | 1M Na_2_SO_4_ | 293 | 17.8 | 0.2 | [2] |
| AgNPS/ CNT | 0.5 M NaOH | 757 | 60.7 | 3.3 | [3] |
| Pure Ag nanoparticle-based | 0.5 M NaOH | 452 | 27.8 | 4.1 | [4] |
| Ag NPS | 1 M NaOH | 423 | 14.04 | 6.41 | [5,6] |
| CPL-AgNPs / CuS | 1M Na_2_SO_4_ | 97 | 34 | 1.4 | **-** |

**
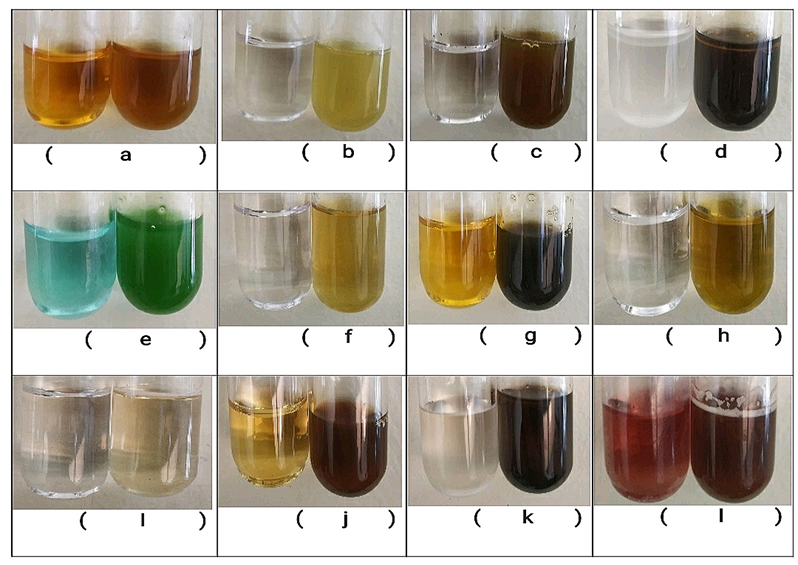
**

**Fig. S1.** Photochemical analysis of CPL extract; a) Alkaloid test; b) anthocyanin test; c) anthroquion test d) carbohydrate test; e) diterpine test; f) flavonoid test; g) glycoside test; h) phenol test; I) phlobatanin test; j) protein test; k), tannin test; l) terpenoid test

|  |  |
| --- | --- |
|  |  |

**Fig. S2** shows the findings of the synthesis of AgNPs using CPL extract at different combinations using UV-visible spectroscopy. AgNO_3_ concentrations (1 mM, 2.5 mM, 5 mM, 7.5 mM, and 10 mM) **(A)**; ratios of AgNO_3_ solution to CPL extract (1*1, 1*2, 1*3, 1*4, and 1*5) **(B);** temperature (4°C, 20°C, 40°C, 60°C) **(C)**; reaction time (0 h, 4 h, 8 h, and 16 h) **(D)**.

| 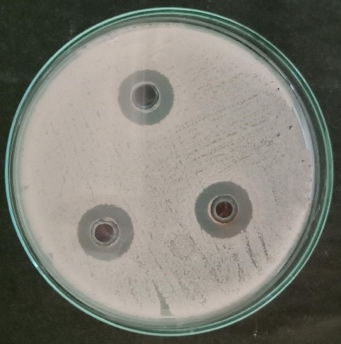**(A)** | 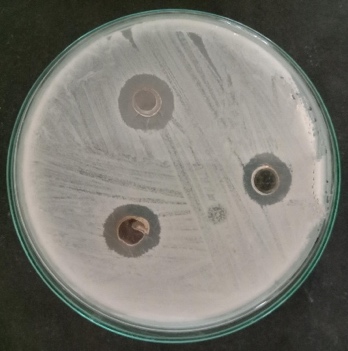**(B)** | 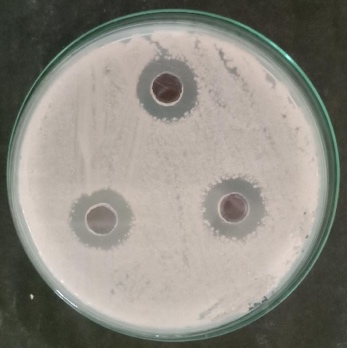**(C)** |
| --- | --- | --- |
| 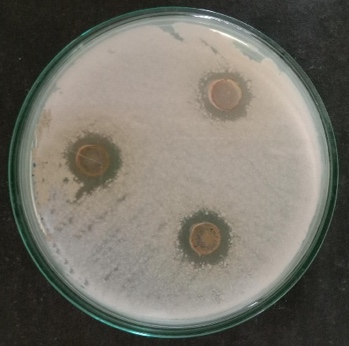**(A-1)** | 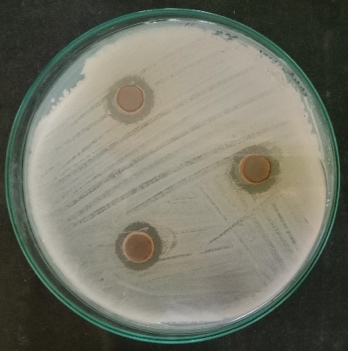**(B-1)** | 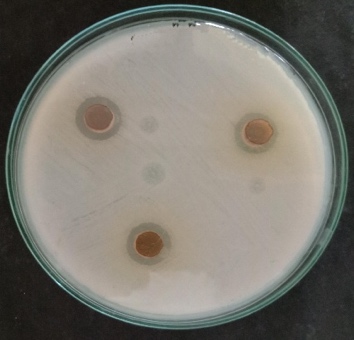**(C-1)** |
| 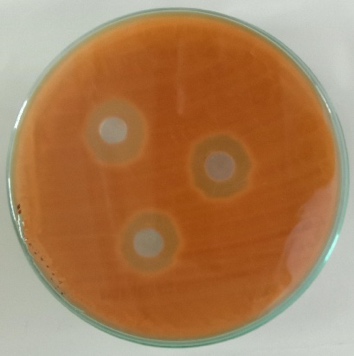**(D)** | 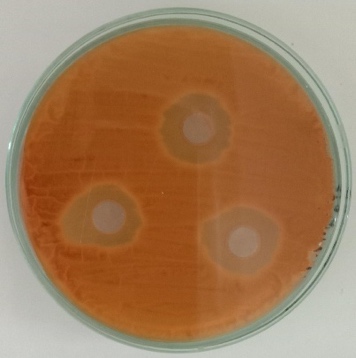**(E)** | 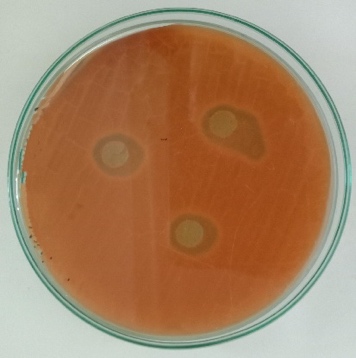**(F)** |
| 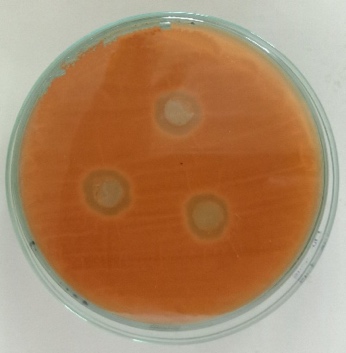**(D-1)** | 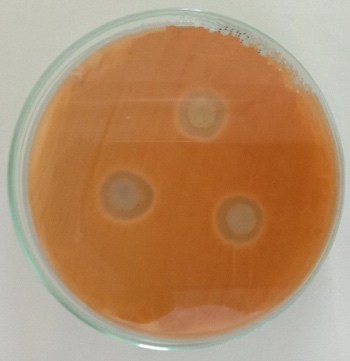**(E-1)** | 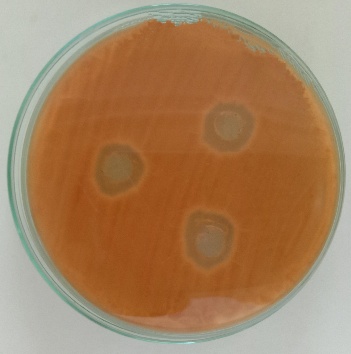**(F-1)** |

**Fig. S3** Antibacterial activity of biosynthesized CPL-AgNPs: antibacterial activity of CPL-AgNPs against *B. subtilis*) (A); *B. megaterium*) (B); *S. flexneri* (C); an antibiotic Penicillin as control (A-1) for *B. subtilis*, for *B. megaterium* (B-1) and *S. flexneri* (C-1); Antifungal activity of biosynthesized CPL-AgNPs: antifungal activity of CPL-AgNPs against *A. niger* (D); *P. crysogenum* (E); *T. viride* (F); an antibiotic as positive control for *A. niger* (D-1); *P. crysogenum* (E-2); *T. viride* (F-2).

**References**

1. Shaikh SB, Waikar MR, Mohite RA, Jadhav SB, Lokhande CD, Pawaskar PN. Carbon-Based Functional Materials for Optical Sensors. In Advanced Functional Materials for Optical and Hazardous Sensing: Synthesis and Applications. Singapore: Springer Nature Singapore. 2023 Nov 3; 27:119-151. <https://doi.org/10.1007/978-981-99-6014-9_6>
2. Shen L, Du L, Tan S, Zang Z, Zhao C, Mai W. Flexible electrochromic supercapacitor hybrid electrodes based on tungsten oxide films and silver nanowires. Chemical communications. 2016; 52:37:6296-309. <https://doi.org/10.1039/C6CC01139J>
3. Pandit B, Devika VS, Sankapal BR. Electroless-deposited Ag nanoparticles for highly stable energy-efficient electrochemical supercapacitor. Journal of Alloys and Compounds. 2017 Dec 5; 726:1295-303. <https://doi.org/10.1016/j.jallcom.2017.08.068>
4. Xia H, Hong C, Shi X, Li B, Yuan G, Yao Q, Xie J. Hierarchical heterostructures of Ag nanoparticles decorated MnO_2_ nanowires as promising electrodes for supercapacitors. Journal of Materials Chemistry A. 2015; 3:3:1216-21. <https://doi.org/10.1039/C4TA05568C>
5. Pandit B, Sankapal BR. Highly conductive energy efficient electroless anchored silver nanoparticles on MWCNTs as a supercapacitive electrode. New Journal of Chemistry. 2017;41(19):10808-14. DOI <https://doi.org/10.1039/C7NJ01792H>
6. Lokhande AC, Babar PT, Karade VC, Jang JS, Lokhande VC, Lee DJ, Kim IC, Patole SP, Qattan IA, Lokhande CD, Kim JH. A viable green route to produce Ag nanoparticles for antibacterial and electrochemical supercapacitor applications. Materials Today Chemistry. 2019 Dec 1; 14:100181-192. https://doi.org/10.1016/j.mtchem.2019.07.003
